# The Transcription Factor ZEB2 Is Required to Maintain the Tissue-Specific Identities of Macrophages

**DOI:** 10.1016/j.immuni.2018.07.004

**Published:** 2018-08-21

**Authors:** Charlotte L. Scott, Wouter T’Jonck, Liesbet Martens, Helena Todorov, Dorine Sichien, Bieke Soen, Johnny Bonnardel, Sofie De Prijck, Niels Vandamme, Robrecht Cannoodt, Wouter Saelens, Bavo Vanneste, Wendy Toussaint, Pieter De Bleser, Nozomi Takahashi, Peter Vandenabeele, Sandrine Henri, Clare Pridans, David A. Hume, Bart N. Lambrecht, Patrick De Baetselier, Simon W.F. Milling, Jo A. Van Ginderachter, Bernard Malissen, Geert Berx, Alain Beschin, Yvan Saeys, Martin Guilliams

**Affiliations:** 1 Laboratory of Myeloid Cell Ontogeny and Functional Specialization, VIB-UGent Center for Inflammation Research, Ghent, Belgium; 2 Department of Biomedical Molecular Biology, Ghent University, Ghent, Belgium; 3 Institute of Infection, Immunity and Inflammation, College of Medical, Veterinary and Life Sciences, University of Glasgow, UK; 4 Data Mining and Modeling for Biomedicine, VIB-UGent Center for Inflammation Research, Ghent, Belgium; 5 Molecular and Cellular Oncology Lab, VIB-UGent Center for Inflammation Research, Ghent, Belgium; 6 Laboratory of Mucosal Immunology and Immunoregulation, VIB Center for Inflammation Research, Ghent, Belgium; 7 Department of Respiratory Medicine, Ghent University, Ghent, Belgium; 8 Laboratory of Molecular Signaling and Cell Death, VIB-UGent Center for Inflammation Research, Ghent, Belgium; 9 Centre d’Immunologie de Marseille-Luminy, Aix Marseille Université, INSERM, CNRS 13288 Marseille, France; 10 MRC Centre for Inflammation Research, University of Edinburgh, The Queen's Medical Research Institute, UK; 11 Mater Research-University of Queensland, Translational Research Institute, Qld 4102, Australia; 12 Myeloid Cell Immunology Lab, VIB-UGent Center for Inflammation Research, Brussels, Belgium; 13 Laboratory of Cellular and Molecular Immunology, Vrije Universiteit Brussel, Brussels, Belgium; 14 Centre d’Immunophénomique, Aix Marseille Université, INSERM, CNRS, 13288 Marseille, France; 15 Department of Applied Mathematics, Computer Science and Statistics, Ghent University, Ghent, Belgium

**Keywords:** Macrophage, Transcription Factor, ZEB2, LXRα, Identity, *Clec4f-cre*, *Fcgr1-cre*

## Abstract

Heterogeneity between different macrophage populations has become a defining feature of this lineage. However, the conserved factors defining macrophages remain largely unknown. The transcription factor ZEB2 is best described for its role in epithelial to mesenchymal transition; however, its role within the immune system is only now being elucidated. We show here that *Zeb2* expression is a conserved feature of macrophages. Using *Clec4f-cre*, *Itgax-cre*, and *Fcgr1-cre* mice to target five different macrophage populations, we found that loss of ZEB2 resulted in macrophage disappearance from the tissues, coupled with their subsequent replenishment from bone-marrow precursors in open niches. Mechanistically, we found that ZEB2 functioned to maintain the tissue-specific identities of macrophages. In Kupffer cells, ZEB2 achieved this by regulating expression of the transcription factor LXRα, removal of which recapitulated the loss of Kupffer cell identity and disappearance. Thus, ZEB2 expression is required in macrophages to preserve their tissue-specific identities.

## Highlights

• ZEB2 is highly expressed across the macrophage lineage
• ZEB2 preserves the tissue-specific identities of macrophages across tissues
• ZEB2 deficient macrophages are outcompeted by WT counterparts
• LXRα is crucial for Kupffer cell identity and is maintained by ZEB2

## Graphical Abstract

**Figure:**
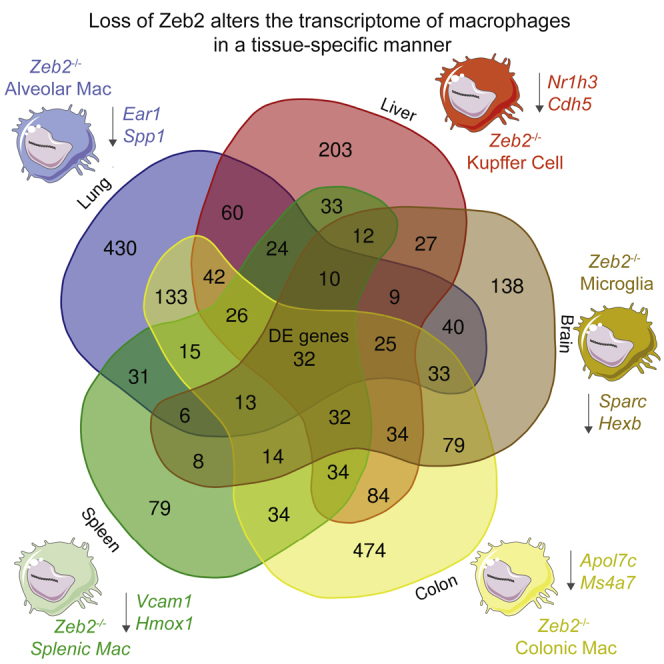

## Teaser

Scott et al. demonstrate that ZEB2 is critical for maintaining the tissue identities of macrophages. Loss of ZEB2 results in tissue-specific changes in different macrophage populations and their subsequent disappearance. In Kupffer cells, ZEB2 maintains LXRα expression, loss of which reproduces the change in Kupffer cell identity and their disappearance.

## Introduction

Most macrophages (macs) arise during embryogenesis from either yolk-sac macs or fetal liver monocytes and self-maintain throughout life in most tissues (Ginhoux and Guilliams, 2016). In a selection of tissues including the heart, gut, and dermis, this self-maintenance is partially abrogated resulting in the continual replenishment of these macs from bone marrow (BM) monocytes (Ginhoux and Guilliams, 2016). In addition, macs across different organs are highly heterogeneous (Gautier et al., 2012, Gosselin et al., 2014, Lavin et al., 2014) and contribute to tissue homeostasis by performing different “accessory functions” in their specific tissues of residence (Okabe and Medzhitov, 2016). Research has recently been focused on understanding the heterogeneity of macs from one tissue to another, but it remains largely unknown if macs also require some conserved factors for their identity, irrespective of their tissue of residence. While high expression of the transcription factor (TF) PU.1 (Monticelli and Natoli, 2017) and dependence on signaling through the colony stimulating factor-1 receptor (CSF1R) (Gow et al., 2014, Hume et al., 1988, Tagliani et al., 2011, Wang et al., 2012) are characteristics of the mac lineage, not much else is known regarding additional conserved TFs that drive and maintain these cells.

Zinc finger E box binding homeobox 2 (ZEB2, SIP1, ZFXH1B) is a TF best known for its role in epithelial to mesenchymal transition (EMT), in which epithelial cells lose their cellular identity and are converted into mesenchymal cells (Brabletz and Brabletz, 2010). EMT transitions are crucial in embryonic development, wound healing, and cancer (De Craene and Berx, 2013). Mice lacking *Zeb2* are embryonic lethal (Higashi et al., 2002), while patients with heterozygous abnormalities in *Zeb2* often develop Hirschsprung’s disease and Mowat-Wilson syndrome (Vandewalle et al., 2009). In the immune system, it has recently been reported that ZEB2 functions to regulate NK cell maturation (van Helden et al., 2015), the terminal differentiation of CD8^+^ effector T cells (Dominguez et al., 2015, Omilusik et al., 2015), and the differentiation and development of pDCs and cDC2s (Scott et al., 2016a, Wu et al., 2016). Additionally, ZEB2 has been suggested to play a role in controlling the fate of the granulocyte-macrophage progenitor (GMP) (Wu et al., 2016). Here, we examined *Zeb2* expression in a variety of mac populations and show that high expression of *Zeb2* is a conserved feature of the mac lineage. Furthermore, we found that loss of ZEB2 in five different macs resulted in the loss of their tissue identities and their subsequent disappearance. More specifically, we found that ZEB2 functions to maintain KC identity, at least in part, by regulating expression of the TF LXRα (*Nr1h3*).

## Results

### *Zeb2* Expression Is Conserved across the Mac Lineage

Although macs represent a highly heterogeneous lineage (Gautier et al., 2012, Lavin et al., 2014, Scott et al., 2016b), we sought here to identify TFs conserved across the mac lineage. To this end, we compiled data from the Immgen Consortium, our previously published studies (Scott et al., 2016b, van de Laar et al., 2016) and data generated during this study. This comparison yielded a list of 67 core mac genes (Figure S1A). Included in this list are genes previously ascribed to the mac lineage including *Fcgr1*, *Mertk*, and *Cd14* (Gautier et al., 2012, Guilliams et al., 2016), as well as the TF *Zeb2*. While this TF has also recently been identified as a core gene in pre-macs (Mass et al., 2016), its precise role within the mac lineage has not been investigated.

### Loss of ZEB2 in KCs and AMs Results in an Altered Phenotype

**Figure 1:**
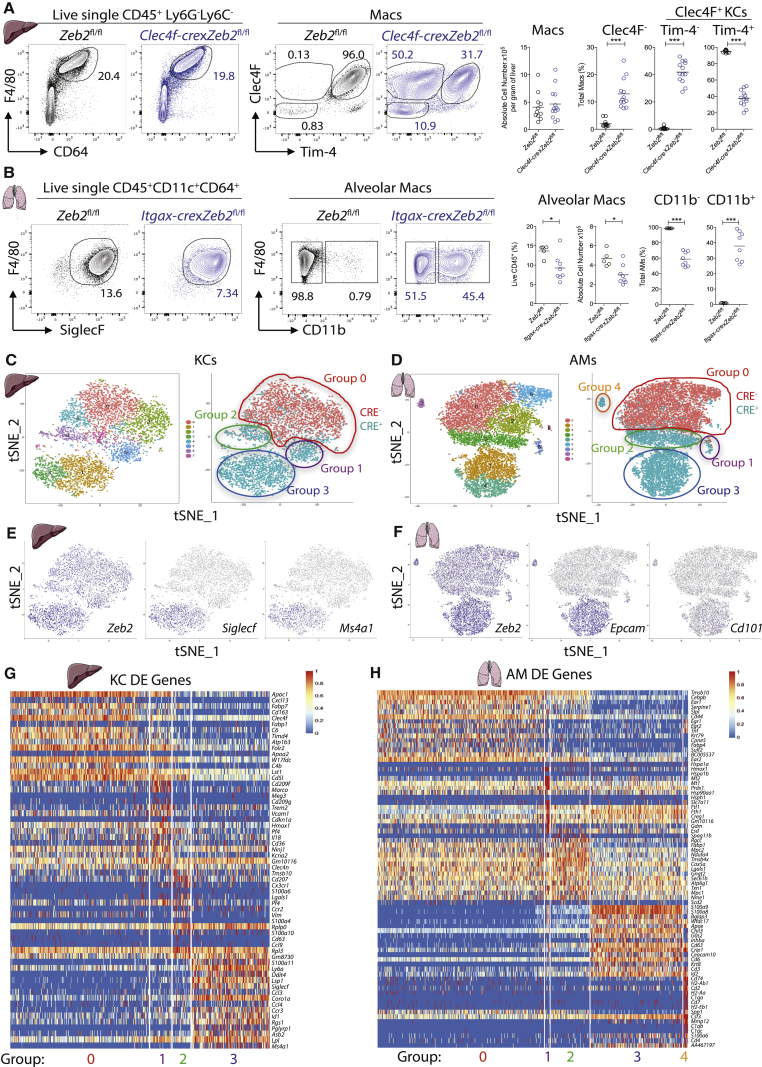
ZEB2 Controls Mac Number and Surface Phenotype (A) Expression of CD64 and F4/80 by live CD45^+^Ly6G^−^Ly6C^-^ liver cells and Clec4F and Tim4 by total liver macs in *Clec4f-cre*x*Zeb2*^fl/fl^ and *Zeb2*^fl/fl^ mice. Absolute number of liver macs per gram of liver and % of total macs expressing Clec4F and Tim4. Data are pooled from four experiments with n = 11–13 per group. ^∗∗∗^p < 0.001 Student’s t test. (B) Expression of SiglecF, F4/80, and CD11b by live CD45^+^CD64^+^CD11c^+^ Lung macs in *Itgax-cre*x*Zeb2*^fl/fl^ and *Zeb2*^fl/fl^ mice. AMs as a percentage of total live CD45^+^ cells, absolute number, and percentage of CD11b^+^ and CD11b^−^ AMs in *Itgax-cre*x*Zeb2*^fl/fl^ or *Zeb2*^fl/fl^ mice. Data are pooled from two experiments with n = 7–8 per group. ^∗^p < 0.05, ^∗∗∗^p < 0.001 Student’s t test. (C and D) t-SNE plot of SC-RNA-seq data of KCs from *Clec4f-cre*x*Zeb2*^fl/fl^ or *Zeb2*^fl/fl^ mice (C) or AMs from *Itgax-cre*x*Zeb2*^fl/fl^ or *Zeb2*^fl/fl^ mice (D), showing clusters, assigned groups, and CRE^−^ (Red) and CRE^+^ (Teal) overlay. (E) tSNE plots showing expression of *Zeb2*, *Siglecf*, and *Ms4a1* in KCs. (F) tSNE plots showing expression of *Zeb2*, *Epcam*, and *Cd101* in AMs. (G and H) Top 15 DE genes per group based on LogFC per group of KCs (G) or AMs (H). See also Figure S1.

Given that *Zeb2*^−/−^ mice are embryonic lethal (Higashi et al., 2002), we utilized CRE-LOX systems to specifically remove *Zeb2* from different mac subsets. Based on *Zeb2* expression (Figure S1A), we first examined the effects of *Zeb2* loss in KCs (higher *Zeb2*) and AMs (lower *Zeb2*). Having recently shown that the C-type lectin, Clec4F, is exclusively expressed by murine KCs (Scott et al., 2016b) and because KCs are poorly targeted by other available CREs, we generated *Clec4f-cre* mice. Crossing these mice to the Rosa26-RFP reporter line revealed that the majority of RFP-expressing cells were CD64^+^F4/80^+^Clec4F^+^Tim4^+^ KCs (Figures S1B–S1E). However, a minor population of B cells, despite lacking expression of Clec4F, were also found to express RFP (Figures S1B–S1E). Despite this minor contamination, we crossed the mice to *Zeb2*^fl/fl^ mice to study the consequences of deleting *Zeb2* in KCs. Analysis of the mac compartment in the liver of *Clec4f-cre*x*Zeb2*^fl/fl^ mice revealed that although there was no significant difference in the absolute number of total CD64^+^F4/80^+^ hepatic macs compared with *Zeb2*^fl/fl^ controls (Figure 1A), there was a difference in their surface phenotype, with *Clec4f-cre*x*Zeb2*^fl/fl^ mice having a reduced population of Clec4F^+^Tim4^+^ KCs and increased populations of Clec4F^+^Tim4^−^ KCs and Clec4F^−^Tim4^−^ macs (Figure 1A). This suggests that ZEB2 might be important for KCs and also highlights the importance of examining tissue-specific mac markers.

As ZEB2 appears to play a role in KCs, we next examined if it also was required by AMs. To remove ZEB2 from AMs, we made use of *Itgax-cre* mice, which efficiently target AMs alongside a number of other CD11c-expressing cells (Durai and Murphy, 2016). By crossing the *Itgax-cre* mice to Rosa26-RFP reporters we confirmed that AMs were efficiently targeted (Figure S1F). Analysis of the total AM population in *Itgax-cre*x*Zeb2*^*fl/fl*^ and *Zeb2*^*fl/fl*^ controls revealed a slight reduction in AMs (Figure 1B). In addition, the loss of *Zeb2* from CD11c-expressing cells also altered the surface phenotype of the remaining AMs with a proportion expressing CD11b in the CRE^+^ mice (Figure 1B).

### *Zeb2*^*+/−*^ Macs Are Present in the Lung and the Liver

To understand how *Zeb2* expression was affecting macs, we performed single-cell RNA sequencing analysis (SC-RNA-Seq) on total KCs (Clec4F^+^CD64^+^F4/80^+^) and total AMs (CD64^+^F4/80^+^SiglecF^+^CD11c^+^) from *Clec4f-cre*x*Zeb2*^fl/fl^ or *Itgax-cre*x*Zeb2*^fl/fl^ mice compared with *Zeb2*^fl/fl^ littermate controls. Following pre-processing of the data using the Marioni pipeline (Lun et al., 2016), poor quality, contaminating, and actively proliferating cells were excluded (Figure S1G) and t-SNE plots with both CRE^−^ and CRE^+^ cells combined for KCs or AMs were generated (Figures 1C and 1D). Next, we determined which cells originated from the CRE^−^ and CRE^+^ mice. This analysis revealed the presence of multiple populations of CRE^+^ cells in both the KCs and AMs (Figures 1C and 1D). To begin to assess what these distinct populations were, we grouped these clusters based on their genotype. For the KCs, this led to the identification of 1 group of CRE^−^ cells (consisting of clusters 0, 2, 4, 7, referred to as group 0) and 3 distinct groups of CRE^+^ cells (cluster 6 = group 1, cluster 5 = group 2 and clusters 1+3 = group 3) (Figure 1C). For the AMs, we identified 1 group of CRE^−^ cells (group 0; clusters 0, 2, 5, 8), one group of mixed CRE^−^ and CRE^+^ cells (group 1 = cluster 6), and three groups of CRE^+^ cells (group 2 = cluster 3, group 3 = clusters 1 + 4 and group 4 = cluster 7) (Figure 1D). Next, we examined *Zeb2* expression between the groups. However, as the *Zeb2*^fl/fl^ construction generates a truncated form of the mRNA possessing a 3’ end it was not possible to determine which cells express full-length or floxed mRNA with the 3’ Assay from 10X Genomics. As such, we were unable to conclude based on *Zeb2* expression if these cells had all efficiently deleted *Zeb2*, but we identified a group of CRE^+^ cells that appeared to have higher *Zeb2* expression in each organ (Figures 1E and 1F – group 3 in KCs and AMs). Thus, we next sought to find markers that could distinguish the different CRE^+^ populations by flow cytometry. To this end, we next determined the differentially expressed (DE) genes between these groups. For the KCs, this generated a list of 224 DE genes for group 0, 180 for group 1, 534 for group 2 and 693 for group 3 (Figure 1G & Table S1) and identified SiglecF and CD20 (*Ms4a1*) to be markers that could potentially be used to distinguish between the groups of CRE^+^ cells (Figure 1E). For the AMs, this analysis identified 821 DE genes for group 0, 312 for group 1, 230 for group 2, 929 for group 3 and 883 in group 4 (Figure 1H & Table S2) and identified CD326 (*Epcam*) and CD101, as two markers which could distinguish between the groups of CRE^+^ cells (Figure 1F).

**Figure 2:**
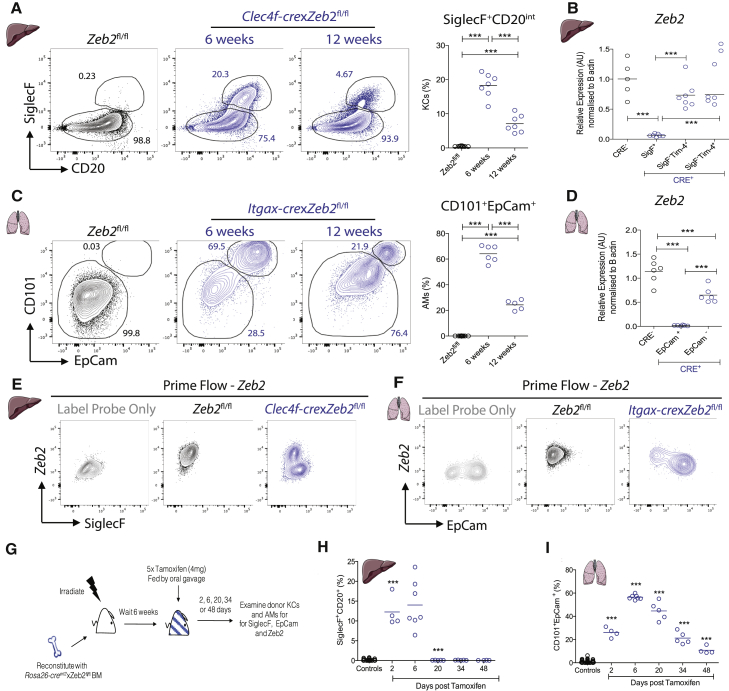
*Zeb2*^−/−^ Macs Are Lost with Time (A) SiglecF and CD20 expression by Clec4F^+^ KCs at 6 and 12 weeks of age compared with *Zeb*2^fl/fl^ controls. Data are from one or two experiments with n = 7–10 per group. ^∗∗∗^p < 0.001 one-way ANOVA with Bonferroni post-test. (B) Relative expression of *Zeb2* mRNA normalized to β-actin as determined by qPCR of sorted SiglecF^+^ and SiglecF^−^ KCs compared with CRE^−^ controls. Data are pooled from one experiment with n = 5–7 per group. ^∗∗∗^p <0.001 one-way ANOVA with Bonferroni post-test. (C) Expression and percentage of EpCam and CD101 by AMs at 6 and 12 weeks of age compared with *Zeb2*^fl/fl^ controls. Data are pooled from one or two experiments with n = 5–11 per group. (D) Relative expression of *Zeb2* mRNA normalized to β-actin as determined by qPCR of sorted EpCam^+^ and EpCam^−^ AMs compared with CRE^−^ control AMs. Data are from one experiment with n = 5–7 per group. (E) Expression of *Zeb2* mRNA and SiglecF in KCs from *Zeb2*^fl/fl^ and *Clec4f-cre*x*Zeb2*^fl/fl^ mice compared with label probe only control. Data are from one experiment with n = 5–6 per group. (F) Expression of *Zeb2* mRNA and EpCam in AMs from *Zeb2*^fl/fl^ and *Itgax-cre*x*Zeb2*^fl/fl^ mice compared with label probe only control. Data are from one experiment with n = 4–5 per group. (G) Schematic of experimental set up. (H) Percentage SiglecF^+^CD20^int^ KCs amongst total CD45.2^+^ KCs and (I) percentage CD101^+^EpCam^+^ AMs amongst total CD45.2^+^ AMs at indicated time points (days) post the last dose of tamoxifen. Data are pooled from two experiments with n = 4–7 per time-point. ^∗∗∗^p < 0.001, one-way ANOVA with Bonferroni post-test. In (H) and (I) each time point is compared to the previous time point and controls are pooled from donor macs from mice administered corn oil and host macs from mice administered tamoxifen. See also Figure S2.

We next examined expression of these markers by flow cytometry. While not expressed by KCs from *Zeb2*^fl/fl^ mice, SiglecF and CD20 were found to be expressed by a proportion of KCs in *Clec4f-cre*x*Zeb2*^fl/fl^ mice at 6 weeks of age (Figure 2A). qRT-PCR analysis for *Zeb2* in SiglecF^+^, SiglecF^−^Tim4^+^ and SiglecF^−^Tim4^−^ KCs (corresponding to group 3, group 1, and group 2, respectively) revealed that SiglecF^+^ KCs had efficiently deleted *Zeb2*, while SiglecF^−^ cells maintained expression of *Zeb2* comparable with KCs isolated from *Zeb2*^fl/fl^ control mice (Figure 2B). Similarly, analysis of EpCam and CD101 expression in AMs from *Itgax-cre*x*Zeb2*^fl/fl^ mice identified two populations, those expressing EpCam and CD101 and those negative for both markers, with only the latter population being observed in AMs from *Zeb2*^fl/fl^ mice (Figure 2C). Again, qRT-PCR analysis determined that only the EpCam^+^CD101^+^ AMs had efficiently deleted *Zeb2* (Figure 2D). As there is no good antibody to detect ZEB2 by flow cytometry, we made use of the prime flow assay, which measures *Zeb2* mRNA expression by flow cytometry to confirm the qRT-PCR analysis. This confirmed that SiglecF^+^ KCs and EpCam^+^ AMs had all efficiently deleted *Zeb2* (Figures 2E and 2F). Genomic PCR on the distinct populations of KCs and AMs identified the SiglecF^−^ KCs and EpCam^-^ AMs as being heterozygous for the *Zeb2* deletion (Figures S2A and S2B), indicating that, for an unknown reason, these cells are able to preserve a copy of *Zeb2*. Returning to the SC-RNA-seq analysis, we could then identify group 0 in each tissue to be *Zeb2*^*+/+*^ macs from the CRE^−^ mice and group 3 in each tissue to represent *bona fide Zeb2*^*-/-*^ macs from the CRE^+^ mice. Group 3 in each tissue was the also the group expressing higher *Zeb2*, suggesting that a feedback mechanism might be in place in the *Zeb2*^*−/−*^ macs, where these cells attempt to increase the expression of the truncated *Zeb2* mRNA. As we have recently shown that Tim4 expression on KCs correlated with the time these cells have spent in the tissue (Scott et al., 2016b), we next defined group 1 KCs which lacked expression of *Siglecf* and expressed *Timd4* as long-lived *Zeb2*^*+/−*^ KCs, while group 2 KCs which lacked expression of *Siglecf* and *Timd4* but which expressed *Cx3cr1* and *Ccr2* were defined as *Zeb2*^*+/−*^ putative moKCs that had recently entered the tissue. In the AMs, the minor population Group 1 contains both CRE^−^ *Zeb2*^+/+^ and some CRE^+^ *Zeb2*^*+/−*^ cells. Ingenuity pathway analysis of the DE genes suggested this minor population has an oxidative stress & unfolded protein response signature, which caused them to fall in a separate cluster (data not shown). Group 2 were identified as *Zeb2*^*+/−*^ cells lacking expression of *Epcam* and *Cd101* and the minor group 4 were (alongside the main group 3) also identified as *Zeb2*^*−/−*^ cells expressing *Epcam* and *Cd101.* Analysis of the DE genes between groups 3 and 4 found that these cells clustered separately from the group 3 *Zeb2*^*−/−*^ cells due to their increased expression of MHCII pathway associated genes (Figure 1H). Thus these might represent cells that arise from monocytes, as increased MHCII expression has been reported on monocyte-derived AMs (van de Laar et al., 2016).

### *Zeb2*^*+/−*^ Macs Outcompete Their *Zeb2*^*−/−*^ Counterparts with Time

Having identified a *Zeb2*^*+/−*^ population of macs amongst both the AMs and KCs in the CRE^+^ mice, we next investigated the maintenance of this population with age. We hypothesized that if *Zeb2* expression was critical for macs, then one would expect that the *Zeb2*^+/−^ population would outcompete the *Zeb2*^−/−^ population with time. Thus, we tracked the presence of the SiglecF^+^CD20^int^*Zeb2*^−/−^ KC and CD101^+^EpCam^+^*Zeb2*^−/−^ AM populations at 6 and 12 weeks of age. We found that both *Zeb2*^*−/−*^ KCs (Figure 2A) and *Zeb2*^*−/−*^ AMs (Figure 2C) are reduced at 12 weeks of age. This reduction in SiglecF^+^ KCs between 6 and 12 weeks was confirmed by confocal microscopy (Figure S2C). Moreover, distinct islands of Clec4F^+^Tim4^+^SiglecF^−^ and Clec4F^+^Tim4^−^SiglecF^−^ KCs were observed at both time-points and were increased in size at 12 weeks. This implied that proliferation of *Zeb2*^*+/−*^ KCs may represent a mechanism by which these cells expand with age. To investigate this, we examined expression of the cell proliferation marker Ki-67 by the different KC populations in *Clec4f-cre*x*Zeb2*^fl/fl^ mice. This analysis showed that while *Zeb2*^*+/+*^ KCs in littermate controls proliferated lowly, SiglecF^−^ *Zeb2*^*+/−*^ KCs from *Clec4f-cre*x*Zeb2*^fl/fl^ mice proliferated significantly more. Conversely, *Zeb2*^*−/−*^ SiglecF^+^ KCs from *Clec4f-cre*x*Zeb2*^fl/fl^ mice were restricted in their ability to proliferate (Figures S2D and S2E). In the lung, Ki-67 staining also revealed that *Zeb2*^*−/−*^ EpCam^+^ AMs did not proliferate to any great extent, while their *Zeb2*^*+/−*^ EpCam^−^ counterparts in *Itgax-cre*x*Zeb2*^fl/fl^ proliferated at significantly increased rates compared with EpCam^−^ *Zeb2*^*+/+*^ AMs in littermate controls (Figures S2F and S2G). Given this reduced proliferation by *Zeb2*^*−/−*^ macs, we next sought to determine whether this was due to a defect in their ability to proliferate. Thus, we administered CSF-1Fc or PBS to *Clec4f-cre*x*Zeb2*^fl/fl^ mice, a procedure that has been described to induce KC proliferation (Gow et al., 2014). *Zeb2*^*-/-*^ KCs proliferated efficiently in response to CSF-1 (Figure S2H) indicating that loss of *Zeb2* does not block the proliferative capacity of macs, but rather may be required for their maintenance.

### Loss of *Zeb2* Leads to Mac Disappearance

To examine the idea that loss of *Zeb2* induces mac disappearance, we generated BM chimeras in which CD45.1^+^ mice were irradiated and reconstituted with congenic CD45.2^+^ *Rosa26-cre*^*ert2*^x*Zeb2*^fl/fl^ BM. Chimeras were made to prevent death of the animals due to *Zeb2* loss in non-hematopoietic cells. 6 weeks later, mice were administered tamoxifen for 5 days by oral gavage to induce CRE-mediated loss of *Zeb2*. KCs and AMs were then examined 2, 6, 20, 34, and 48 days after the last dose of tamoxifen and expression of CD101 and EpCam (Lung AMs) or SiglecF and CD20 (Liver KCs) in donor-derived CD45.2 cells was assessed (Figure 2G). Controls include both CD45.2 cells from control mice that were not treated with tamoxifen and host CD45.1^+^ WT cells from mice treated with tamoxifen (Figures 2H and 2I). In the liver, 12.27% ± 3.88% of donor-derived KCs expressed SiglecF and CD20 2 days post the last dose of tamoxifen and this rose modestly at day 6. 20 days post the last dose of tamoxifen, SiglecF^+^CD20^+^ KCs could no longer be detected in the liver implying that the *Zeb2*^−/−^ KCs had disappeared (Figure 2H). This disappearance of *Zeb2*^−/−^ KCs was confirmed by the PrimeFlow assay, as by day 20 all *Zeb2*^−/−^ KCs were lost (Figure S2I). In the lung, 2 days post administration of tamoxifen 26.1% ± 4.23% of CD45.2 donor AMs expressed CD101 and EpCam. This further increased to a maximum of 56.4% ± 2.02% six days post the last dose of tamoxifen. At all subsequent time points examined this dropped continuously reaching 10.78% ± 3.25% at day 48 (Figure 2I). This disappearance of *Zeb2*^−/−^ AMs was also confirmed using the PrimeFlow assay (Figure S2J). Taken together, these results demonstrate that ZEB2 is strictly required for the continued presence of macs in tissues and suggest that loss of ZEB2 may result in impaired mac maintenance.

### Loss of *Zeb2* from KCs but Not AMs, Results in Their Replenishment from BM

**Figure 3:**
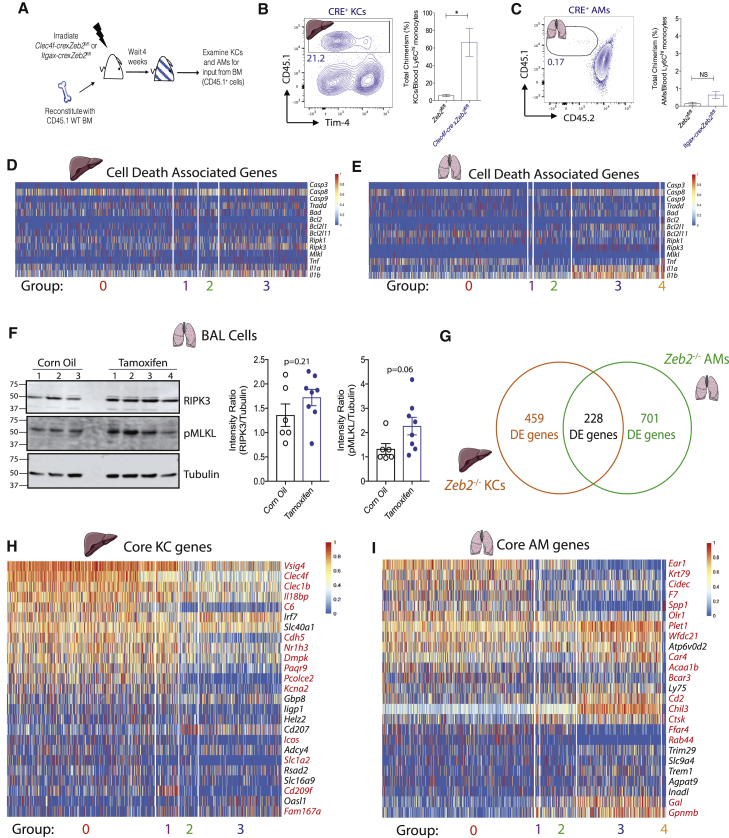
ZEB2 Controls Tissue Identity of KCs and AMs (A) Schematic of experimental set up. (B) Expression of CD45.1 (donor) and Tim4 in total Clec4F^+^ KCs in *Clec4f-cre*x*Zeb2*^fl/fl^ chimeras. Percentage of total chimerism of KCs in Zeb2^fl/fl^ and *Clec4f-cre*x*Zeb2*^fl/fl^ mice. Data are pooled from two experiments with n = 6–10 per group.^∗∗∗^p < 0.001 Student’s t-test. (C) Expression of CD45.1 (donor) and CD45.2 (host) in total lung AMs in *Itgax-cre*x*Zeb2*^fl/fl^ mice. Percentage of total chimerism of AMs in *Zeb2*^fl/fl^ and *Itgax-cre*x*Zeb2*^fl/fl^ mice. Data are pooled from two experiments with n = 5–8 per group. NS; non-significant. Student’s t-test. Percentage total chimerism calculated as ratio over the chimerism in blood Ly6C^hi^ monocytes in the same mouse. (D and E) Heatmap of expression of cell death-associated genes per group of KCs (D) or AMs (E) from SC-RNA-seq data. (F) Representative western blots (n = 2) for RIPK3, pMLKL, and Tubulin expression by total BAL cells isolated from CD45.1 mice that were irradiated (8 Gy) and reconstituted with *Rosa26-cre*^*ert2*^x*Zeb2*^fl/fl^ BM. 33 weeks post reconstitution, mice were fed 5 mg tamoxifen or corn oil as a control for 5 days. 27 days after the last dose, mice were sacrificed and BAL fluid isolated. BAL fluid from 3 or 4 mice was pooled per replicate and 150,000 cells were used per lane. Ratio of band intensity was calculated using ImageJ. Data are pooled from two experiments with n = 6–8 per group. Student’s t-test was used to calculate indicated p values. (G) Venn diagram showing DE genes specific to *Zeb2*^−/−^ KCs (group 3), *Zeb2*^−/−^ AMs (group 3), or shared between both mac populations. (H and I) Heatmap showing expression of top core genes across KC (H) or AM (I) groups from SC-RNA-seq data. Genes in red are significantly differentially expressed. See also Figure S3.

We next investigated whether the *Zeb2*^−/−^ macs were being replenished from the BM or if mac numbers were maintained solely by local proliferation of *Zeb2*^+/−^ counterparts. To examine this, we generated partially-protected chimeras, in which *Clec4f-cre*x*Zeb2*^fl/fl^, *Itgax-cre*x*Zeb2*^fl/fl^ mice or *Zeb2*^fl/fl^ littermate controls were irradiated with their livers or lungs protected and reconstituted with congenic CD45.1 WT BM (Figure 3A). 4 weeks later, we examined the proportion of CD45.1^+^ cells within the blood monocytes and KCs in the liver (defined as Clec4F^+^) or AMs in the lung (defined as CD11c^+^SiglecF^+^). As the mice were partially protected from irradiation, the animals were between 30%–50% chimeric (calculated by examining chimerism in blood Ly6C^hi^ monocytes). Comparison of the chimerism between the blood monocytes and liver KCs found that KCs were chimeric (Figure 3B); however, lung AMs displayed very low chimerism (Figure 3C). To further investigate how *Zeb2*^−/−^ macs were being lost and replaced by *Zeb2*^+/−^ counterparts, we next questioned whether the macs were dying in the absence of ZEB2. We examined expression of a number of genes associated with distinct cell death pathways in our SC-RNA-Seq analysis. Although a number of these genes were either not expressed or their expression was not altered in *Zeb2*^−/−^ macs, we did observe that *Ripk3*, *Il1a*, and *Il1b* were upregulated in *Zeb2*^−/−^ KCs and AMs, suggesting that the loss of *Zeb2* might result in mac death by necroptosis (Figures 3D and 3E). Moreover, we evaluated the expression of RIPK3 and phosphorylated MLKL (pMLKL) by cells recovered from bronchioalveolar lavage (BAL) fluid from the *Rosa26-cre*^*ert2*^*xZeb2*^*fl*/fl^ chimeras 27 days post the last dose of tamoxifen and compared them to chimeras which received corn oil as a control. Total BAL cells were used to prevent induction of cell death during the extensive enzymatic digestions required to isolate macs from tissues. This analysis revealed a trend (p = 0.06) towards increased pMLKL (Figure 3F). Taken together, our results demonstrate that ZEB2 is critical for the maintenance of KCs and AMs, with *Zeb2*^−/−^ macs being lost from the tissue with time. Furthermore, it suggests that *Zeb2*^−/−^ macs might be lost through necroptotic cell death.

### *Zeb2* Controls Tissue-Specific Identity of Lung and Liver Macs

Having identified the distinct populations of KCs and AMs present in the SC-RNA-seq data, we next examined the mechanism through which loss of *Zeb2* induces the mac disappearance. As loss of *Zeb2* affected both KCs and AMs, we first looked for the DE genes that were conserved between both mac populations. However, this demonstrated that the majority of DE genes were unique to either the KC (459 DE genes) or AM (701 DE genes) population (Figure 3G). As the gene-expression profiles of different tissue macs have been shown to be highly heterogeneous and ZEB2 is known for its role in altering cellular identities in EMT, we next hypothesized that ZEB2 might control the tissue-specific identities of the different mac populations, with its loss rendering the macs less suited to their niche resulting in their subsequent loss. To examine this, we investigated how the core KC and AM transcriptional profile changed in the absence of ZEB2. As additional mac populations have been sequenced since the core profiles of these two macs were described (Gautier et al., 2012, Scott et al., 2016b), we first redefined these profiles. Thus, we compared the transcriptional profile of AMs, KCs, microglia, peritoneal macs, colonic macs (CMs), and splenic red pulp macs (SMs) (Lavin et al., 2014) and identified the genes specific to the KCs and AMs (Figure S3). As there was considerable overlap between the transcriptional profiles of KCs and SMs, SMs were excluded when defining the core profile of KCs and vice versa. Furthermore, as these core lists were defined on the basis of bulk RNA seq data, we performed an additional control whereby to be a core gene, it must be expressed in at least 20% of our *Zeb2*^fl/fl^ KCs and AMs profiled by SC-RNA-seq (Figure S3). This was required because previously reported core mac gene lists contained genes expressed by contaminating cells (Lynch et al., 2018). We next compared how expression of the top core genes were altered upon loss of ZEB2 and found that there were numerous changes to the core profiles of both mac populations with 60% of the KC tissue-specific genes and 72% of the AM tissue-specific genes affected by the loss of *Zeb2* (Figures 3H and 3I), suggesting ZEB2 might play a role in maintaining the tissue-specific identities of these macs.

### Loss of KC Identity in Absence of ZEB2 Is in Part Due to the Loss of LXRα

**Figure 4:**
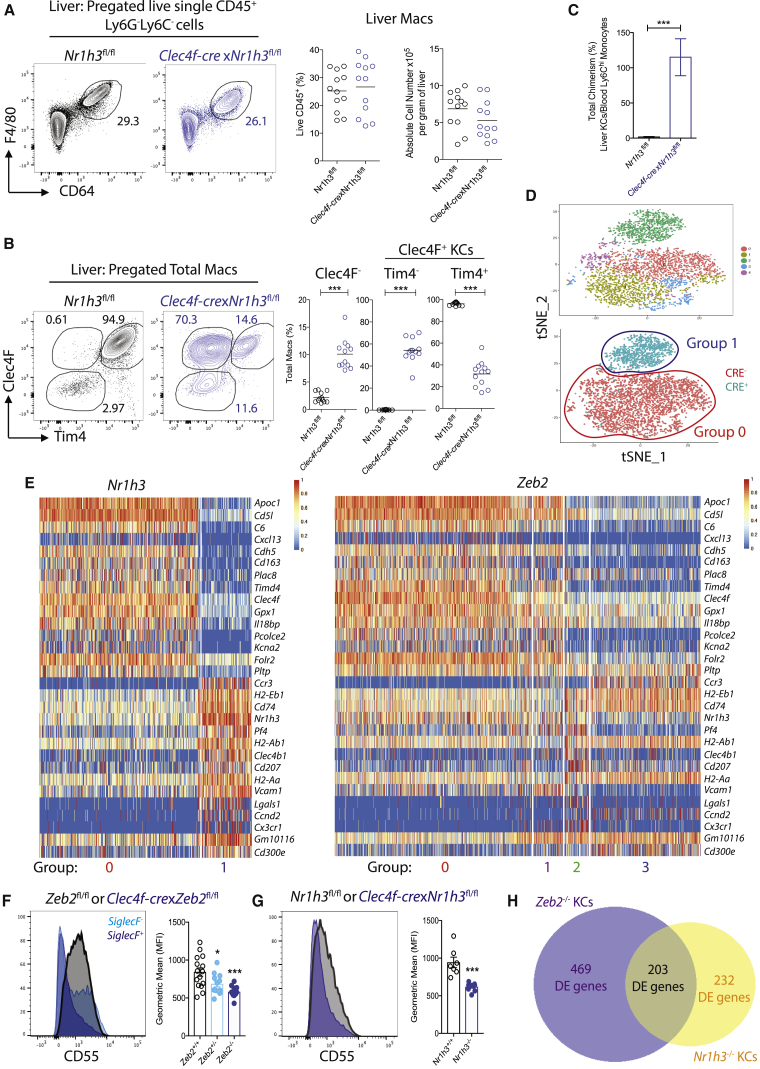
Loss of LXRα from KCs Recapitulates Main Features of *Zeb2*^−/−^ KCs (A) Expression of CD64 and F4/80 by live CD45^+^Ly6G^−^Ly6C^−^ liver cells in *Clec4f-cre*x*Nr1h3*^fl/fl^ and *Nr1h3*^fl/fl^ mice. Liver macs as a percentage of total live CD45^+^ cells and absolute number per gram of liver. (B) Expression of Clec4F and Tim4 by total liver macs in *Clec4f-cre*x*Nr1h3*^fl/fl^ and *Nr1h3*^fl/fl^ mice and percentage of total macs expressing Clec4F and Tim4. Data are pooled from two experiments with n = 12 per group. ^∗∗∗^p < 0.001 Student’s t test. (C) Percentage total chimerism of total Clec4F^+^ KCs in *Nr1h3*^fl/fl^ and *Clec4f-cre*x*Nr1h3*^fl/fl^ mice. Data are pooled from two experiments with n = 6–8 per group. ^∗∗∗^p < 0.001; Student’s t-test. (D) t-SNE plot of SC-RNA-Seq data from KCs from *Clec4f-cre*x*Nr1h3*^fl/fl^ and *Nr1h3*^fl/fl^ mice, showing clusters, assigned groups and CRE^−^ (Red) and CRE^+^ (Teal) overlay. (E) Heatmaps showing top DE genes (15 downregulated, 15 upregulated) based on LogFC in KCs with loss of LXRα and expression of the same genes by the indicated groups of KCs from *Zeb2*^fl/fl^ and *Clec4f-cre*x*Zeb2*^fl/fl^ mice. (F and G) Histogram and MFI of CD55 expression in (F) *Zeb2*^+/+^ (*Zeb2*^fl/fl^), SiglecF^+^*Zeb2*^−/−^ KCs, and SiglecF^−^*Zeb2*^+/−^ KCs from *Clec4f-cre*x*Zeb2*^fl/fl^ mice and (G) Nr1h3^+/+^ (*Nr1h3*^fl/fl^) and Nr1h3^−/−^ KCs from *Clec4f-cre*x*Nr1h3*^fl/fl^ mice. (H) Venn diagram showing DE genes specific to *Zeb2*^−/−^ KCs, *Nr1h3*^−/−^ KCs, or shared between both mac populations. See also Figure S4.

We next examined the mechanism through which ZEB2 could control mac tissue-specific identities. For this, we focused on the KCs. The tissue-specific identity of macs has been proposed to be controlled by a small set of tissue-specific TFs (Lavin et al., 2014). *Nr1h3* (encoding LXRα) was reported to be highly expressed by KCs (Mass et al., 2016) and was among the list of DE core KC identity genes in KCs lacking *Zeb2* (Figure 3H). Thus, we hypothesized, that ZEB2 might control KC identity by regulating LXRα expression. LXRα was previously reported to be dispensable for KC development and survival, but this was based solely on F4/80 and CD68 expression (A-Gonzalez et al., 2013). Therefore, we decided to revisit the effects of loss of *Nr1h3* on KCs. We crossed *Nr1h3*^fl/fl^ mice with *Clec4f-cre* mice generating *Clec4f-cre*x*Nr1h3*^fl/fl^ mice. This confirmed that loss of *Nr1h3* did not affect the proportion or absolute number of total hepatic CD64^+^F4/80^+^ macs (Figure 4A). However, as for the loss of *Zeb2*, it altered the proportions of cells expressing Clec4F and Tim4 (Figure 4B). Protected chimeras demonstrated that in the absence of *Nr1h3*, KCs were being replaced from a BM source (Figure 4C). As these data suggest that ZEB2 might function to control KC identity through maintaining LXRα expression, we next determined whether the effect of *Zeb2* loss on the KCs transcriptome might reflect loss of LXRα-dependent genes. Thus, we performed SC-RNA-seq of KCs from *Clec4f-cre*x*Nr1h3*^fl/fl^ mice and *Nr1h3*^fl/fl^ littermate controls. Following the same pre-processing as above (Figure S1G), we identified 2 main groups of KCs in the t-SNE plot of CRE^−^ and CRE^+^ cells. Group 0 consisted of *Nr1h3*^+/+^ KCs from the CRE^−^ mice and group 1 consisted of *Nr1h3*^−/−^ KCs from the CRE^+^ mice (Figure 4D). Mice lacking only one copy of LXRα in their KCs (*Clec4f-cre*x*Nr1h3*^fl/+^) did not display a similar phenotype to *Clec4f-cre*x*Nr1h3*^fl/fl^ mice, suggesting no obvious effect of *Nr1h3* haploinsufficiency on KCs (Figure S4A). Analysis of the DE genes between *Nr1h3*^+/+^ KCs and *Nr1h3*^−/−^ KCs identified 451 DE genes (Figure 4E and Table S3) and many of these DE genes were also core KC genes including *Cdh5*, *Pcolce2*, *Kcna2*, *C6*, and *Il18bp* (Figure 3H) and were similarly lost upon loss of *Zeb2* (Figure 4E). Moreover, we were able to confirm this downregulation in both *Zeb2*^*−/−*^ and *Nr1h3*^*−/−*^ KCs by flow cytometry (Figures 4F and 4G) or qRT-PCR (Figures S4B and S4C). As loss of ZEB2 and LXRα led to replacement of the KCs from the BM, we noticed that a number of the DE genes were also related to origin of the KCs. To remove any DE genes associated with mac origin and hence only examine DE genes resulting from the loss of LXRα or ZEB2, we used our previously published data (Scott et al., 2016b) and identified any DE genes between moKCs from KC-DTR mice 15 days post treatment with DT and embryonic KCs (Figure S4D). Comparison of the overlap between the remaining non-origin related DE genes associated with the *Zeb2*^*−/−*^ and *Nr1h3*^*−/−*^ KCs identified that 203 of the 435 DE genes in *Nr1h3*^*−/−*^ KCs were conserved in both datasets (Figure 4H), including many of the liver-specific core KC genes. Crucially, while there is overlap between the two genotypes, it is not 100%, indicating that loss of LXRα is not solely responsible for all the DE genes identified in *Zeb2*^*−/−*^ KCs. Nonetheless, loss of LXRα is sufficient to recapitulate the loss of KC identity and the disappearance and replenishment of KCs by BM cells observed in *Clec4f-cre*x*Zeb2*^fl/fl^ mice. Taken together, these data demonstrate that ZEB2 controls KC identity at least in part by regulating LXRα expression.

### ZEB2 Functions across the Mac Lineage to Maintain the Tissue-Specific Identities

**Figure 5:**
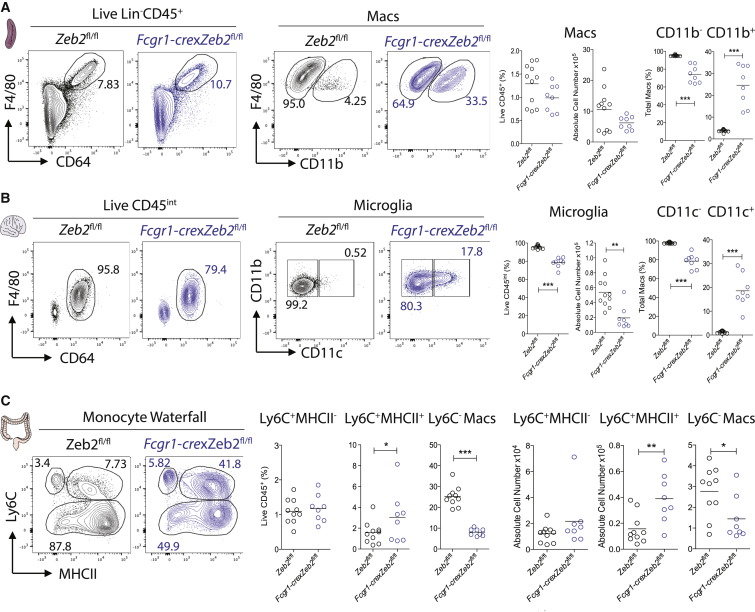
Loss of ZEB2 Affects Mac Phenotype and/or Number across Tissues (A) Expression of CD64, F4/80 and CD11b by live CD45^+^Ly6G^-^CD64^+^Ly6C^−^MHCII^−^ SMs in *Fcgr1-cre*x*Zeb2*^fl/fl^ and *Zeb2*^fl/fl^ mice. SMs as a percentage of total live CD45^+^ cells, absolute number and percentage of CD11b^+^ and CD11b^−^ SMs in *Fcgr1-cre*x*Zeb2*^fl/fl^ or *Zeb2*^fl/fl^ mice. (B) Expression of CD64, F4/80, CD11c, and CD11b by live CD45^int^ microglia in *Fcgr1-cre*x*Zeb2*^fl/fl^ and *Zeb2*^fl/fl^ mice. Microglia as a percentage of total live CD45^+^ cells, absolute number and percentage of CD11c^+^ and CD11c^−^ microglia in *Fcgr1-cre*x*Zeb2*^fl/fl^ or *Zeb2*^fl/fl^ mice. (C) Expression of Ly6C and MHCII (monocyte waterfall) by live CD45^+^CD11b^+^Ly6G^−^SiglecF^−^ non cDCs in *Fcgr1-cre*x*Zeb2*^fl/fl^ and *Zeb2*^fl/fl^ mice. Percentage of live CD45^+^ and absolute number of Ly6C^+^MHCII^−^, Ly6C^+^MHCII^+^, and Ly6C^−^ Macs in *Fcgr1-cre*x*Zeb2*^fl/fl^ or *Zeb2*^fl/fl^ mice. Data are pooled from two experiments with n = 8–11 per group. ^∗^p < 0.05, ^∗∗^p < 0.01, ^∗∗∗^p < 0.001 Student’s t test. See also Figure S5.

We next investigated if ZEB2 was required across the mac lineage. To this end, we crossed the *Zeb2*^fl/fl^ mice to the *Fcgr1-cre* mice recently generated by Bernard Malissen (Figure S5). *Fcgr1-cre*xRosa26-RFP mice revealed that SMs, microglia, and CMs were efficiently targeted with this CRE (Figures S5A–S5D). However, a number of other immune cells are also targeted in these mice. This includes CD64^−^ B cells, T cells, cDC1s, and cDC2s (Figure S5A). *Fcgr1-cre*x*Zeb2*^fl/fl^ mice had no change in the proportion or number of the total SM population defined as Lin^−^CD64^+^F4/80^+^ (Figure 5A), but a proportion of these macs gained expression of CD11b in the absence of *Zeb2* (Figure 5A). In addition, we observed a reduction in absolute number of microglia (Figure 5B) and *Zeb2*^*−/−*^ microglia were found to upregulate their expression of CD11c (Figure 5B). To examine whether ZEB2 also functions in the maintenance of BM monocyte-derived macs, we next examined whether *Zeb2* was also required in CMs which are constantly replaced by BM monocytes during adulthood along a trajectory dubbed the “monocyte-waterfall” (Bain et al., 2013, Bain et al., 2014, Tamoutounour et al., 2012). Analysis of the monocyte-waterfall in *Fcgr1-cre*x*Zeb2*^fl/fl^ mice identified a reduction in the proportion and number of mature CMs alongside an increase in the proportion and number of Ly6C^+^MHCII^+^ transitioning monocytes (Figure 5C).

**Figure 6:**
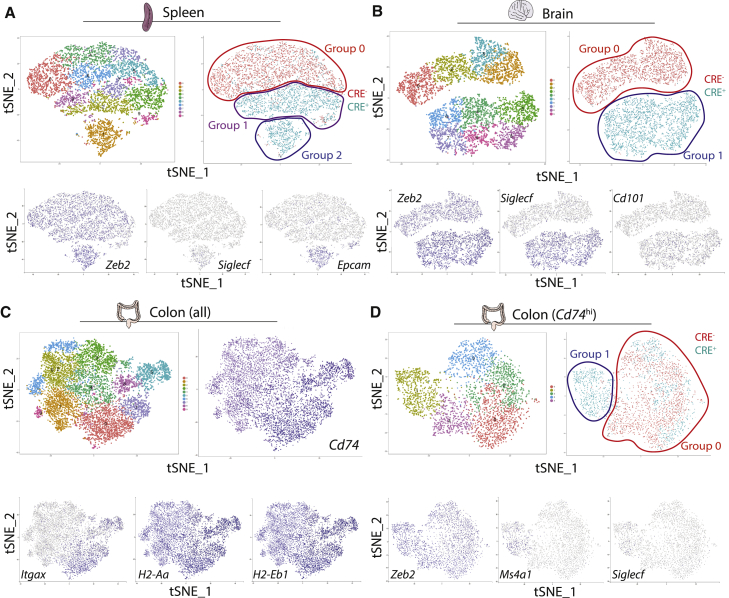
Loss of *Zeb2* Results in Altered Transcriptome across Mac Lineage (A and B) t-SNE plot of SC-RNA-seq data from SMs (A) and microglia (B) sorted from *Fcgr1-cre*x*Zeb2*^fl/fl^ and *Zeb2*^fl/fl^ mice, showing clusters of macs, assigned groups and CRE^−^ (Red) and CRE^+^ (Teal) overlay and expression of indicated genes. (C) t-SNE plot of SC-RNA-seq data from total CMs from *Fcgr1-cre*x*Zeb2*^fl/fl^ and *Zeb2*^fl/fl^ mice, showing clusters of macs and *Cd74*, *Itgax*, *H2-Aa*, and *H2-Eb1* expression. (D) t-SNE plot of SC-RNA-seq data from *Cd74*^hi^ CMs from *Fcgr1-cre*x*Zeb2*^fl/fl^ and *Zeb2*^fl/fl^ mice, showing clusters, assigned groups and CRE^−^ (Red) and CRE^+^ (Teal) overlay and expression of indicated genes. See also Figure S6.

To determine whether these changes reflected altered tissue-specific identities of these macs, we next performed SC-RNA-seq analysis. Following the same pre-processing as above (Figure S1G), we used expression of *Zeb2*, *Ms4a1*, *Siglecf*, *Cd101*, or *Epcam* to identify *Zeb2*^*−/−*^ macs in the spleen, brain, and colon (Figure 6). Note that, as was observed for the lung and liver, the *Zeb2*^*−/−*^ macs in the spleen, brain, and colon expressed higher *Zeb2*, again suggesting a feedback mechanism in the *Zeb2*^*−/−*^ macs (Figure 6). We identified three main groups of cells in the SMs (group 0; *Zeb2*^*+/+*^ macs from the CRE^−^ mice, group 1; presumably *Zeb2*^*+/−*^ macs from the CRE^+^ mice clustering close to the *Zeb2*^*+/+*^ macs from the CRE^−^ mice and group 2; *Zeb2*^*−/−*^ macs from the CRE^+^ mice clustering separately and expressing higher *Zeb2*, *Siglecf*, and *Epcam*) (Figure 6A). In addition, we identified 2 groups of cells in the microglia (group 0; *Zeb2*^*+/+*^ macs from the CRE^−^ mice and group 1; *Zeb2*^*−/−*^ macs from the CRE^+^ mice expressing higher *Zeb2*, *Siglecf*, and *Cd101*) (Figure 6B). While we performed the SC-RNA-seq analysis on total CMs (Figure 6C), we found these could be divided into two main groups of cells, those expressing high *Cd74* (coding for the MHCII-associated invariant chain), *H2-Aa*, *H2-Eb1*, and *Itgax* (coding for CD11c) and those expressing low *Cd74* (Figure 6C). As all the *Zeb2*^*−/−*^ cells (identified by higher *Zeb2*, *Ms4a1*, and *SiglecF*) expressed high *Cd74* (Figures 6D) and as the gene-expression profiles of the *Cd74*^*hi*^ and *Cd74*^*lo*^ *Zeb2*^+/+^ subsets of CMs from CRE^-^ mice were distinct (Figure S6), we chose to focus our analysis on the comparison between *Cd74*^*hi*^ *Zeb2*^+/+^ macs and *Cd74*^*hi*^ *Zeb2*^*−/−*^ macs (Figure 6D). Within *CD74*^*hi*^ macs, we identified 2 main groups of cells (group 0; containing a mix of *Zeb2*^*+/+*^ macs from the CRE^−^ mice and presumably *Zeb2*^*+/−*^ macs from the CRE^+^ mice clustering together, and group 1; containing *Zeb2*^*−/−*^ macs from the CRE^+^ mice expressing higher *Zeb2*, *Ms4a1*, and *SiglecF*) (Figure 6D).

**Figure 7:**
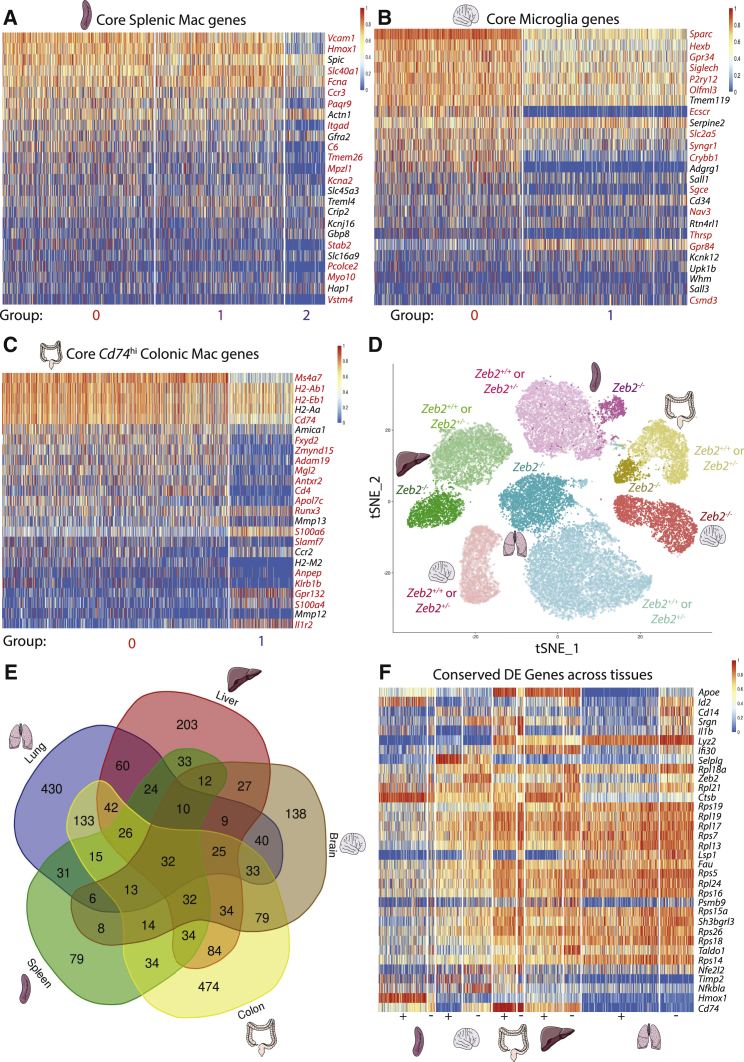
Loss of ZEB2 Results in Loss of Mac Tissue-Specific Identity across Tissues (A–C) Heatmap showing expression of top core SM (A), microglia (B), or *Cd74*^hi^ CM (C) genes across indicated groups from SC-RNA-Seq analysis. Genes in red are significantly differentially expressed. (D) t-SNE showing all macs sequenced by SC-RNA-seq from the indicated five tissues. *Zeb2*^−/−^ macs are shown in bold color, *Zeb2*^+/+^ or *Zeb2*^+/−^ macs are shown in faded color. Open circles represent cells arising from CRE^−^ mice while filled circles are those isolated from CRE^+^ mice (*Fcgr1-cre*, *Itgax-cre*, or *Clec4f-cre*). (E) Venn Diagram detailing overlap of DE genes between all five tissue mac populations in the absence of *Zeb2*. (F) Heatmap showing 32 DE genes conserved across all five tissue mac populations. See also Figure S7.

To examine the effects of loss of ZEB2 on mac identity, we next determined the core profiles of these macs as described above (Figures S7A–S7C) and examined the expression of these identity genes in the *Zeb2*^*−/−*^ macs. As for the liver and lung, this revealed that the core profiles of the different macs were altered in the absence of *Zeb2* (60% in SMs and microglia and 76% in CMs; Figures 7A–7C, with indicated groups from Figure 6).

Consistent with the data from the liver and lung (Figures 2G–2I), *Zeb2*^*−/−*^ SMs were also found to disappear in mice in which *Zeb2* loss was induced by tamoxifen administration (Figures S7D and S7E). The brain and colon were unfortunately not assessed but the conserved effects in the liver, lung, and spleen coupled with the reduced population of microglia and increased turnover of CMs strongly suggests that loss of *Zeb2* induces mac disappearance across tissues.

To further confirm that the loss of ZEB2 results in tissue-specific changes, we examined the overlap between the DE genes in the 5 tissues (Tables S1, S2, S4, S5 and S6) and generated a single t-SNE file containing all *Zeb2*^+/+^ and *Zeb2*^*−/−*^ macs from the 5 organs (Figure 7D). This demonstrated that loss of *Zeb2* did not direct the cells from each tissue along a single component in the tSNE plot, suggesting the changes were predominantly tissue-specific. Additionally, we found that the majority of DE genes were specifically altered in only one of the 5 tissues (Figure 7E) with only 32 DE genes being shared by all tissues (Figures 7E and 7F). Taken together, these data highlight that loss of *Zeb2* has a striking tissue-specific effect on mac identity.

## Discussion

TFs are at the core of lineage specification and commitment through regulation of gene expression. TFs can function at various stages in a cell, during development and/or in the maintenance of the terminally-differentiated cells, as well as in controlling specific cellular functions. While a number of TFs have recently been identified in specific mac populations including ID3 in KCs (Mass et al., 2016), PPARγ in AMs (Schneider et al., 2014) and GATA6 in peritoneal macs (Lavin et al., 2014, Okabe and Medzhitov, 2014), the TFs governing the entire mac lineage aside from PU.1 remain to be fully investigated. Here we report that *Zeb2* is highly expressed in macs across tissues. In addition, *Zeb2* has been reported to already be expressed in the embryonic pre-macs (Mass et al., 2016). Our data indicate that ZEB2 was required to maintain the cellular identity of macs with its loss leading to their disappearance from all tissues studied. Therefore, just as ZEB2 controls cell identity in EMT (Brabletz and Brabletz, 2010) it is essential for the maintenance of mac identities across tissues.

SC-RNA-seq analysis of the different mac populations revealed that *Zeb2* was often not efficiently deleted in all macs using the three distinct CRE models we employed, with *Zeb2*^+/−^ populations being observed in all organs except the brain. The mechanism by which some *Zeb2*^+/−^ macs retain one unfloxed *Zeb2* allele remains unclear and requires further investigation. *Zeb2*^*−/−*^ macs were distinguished from *Zeb2*^+/−^ counterparts within the same mouse on the basis of their phenotype. KCs expressed SiglecF and CD20 (encoded by *Ms4a1*) following loss of *Zeb2*, while AMs expressed CD101 and EpCam. These markers, although not conserved between all the mac populations, were conserved in a number of the populations and with *Zeb2*-deficient cDC2s (Scott et al., 2016a) suggesting that ZEB2 expression might be linked with the repression of a set of surface receptors. However, it was not through these markers that we first determined an effect of loss of ZEB2 expression in KCs and AMs. Rather, we noted a loss of Tim4 expression in Clec4F^+^ KCs and an increase in Clec4F^−^ macs within total F4/80^+^CD64^+^ hepatic macs, an increase in CD11b expression in AMs and SMs, an increase in CD11c in microglia and an increased turnover rate of CMs. This highlights the importance of looking at tissue-specific mac markers and not just F4/80 or CD64 when examining different mac populations.

In terms of understanding mac ontogeny, we recently proposed the mac niche hypothesis (Guilliams and Scott, 2017). We suggested that niche availability and niche accessibility would be the two main factors determining mac ontogeny. Loss of ZEB2 within mature macs induces mac disappearance, which creates niche availability. We found that these lost macs are replaced (in part) by cells of BM origin (likely BM monocytes) in the liver but not to any great extent in the lung. It is worth noting, however, that we do see a small population of MHCII-expressing AMs in the SC-RNA-seq analysis and found few BM-derived cells in the chimeras, indicating that a minor fraction of AMs could be replaced by monocytes. This major replacement of KCs but minor replacement of AMs is in line with our niche hypothesis (Guilliams and Scott, 2017) as only the liver mac niches are accessible to progenitors circulating in the blood as liver KCs reside in the bloodstream of the liver sinusoids while the AM niches are protected by the lung epithelial barrier. However, we only see a small reduction in total AM cell number following loss of ZEB2 despite there being very limited replenishment from the BM. This is because the *Zeb2*^+/−^ AMs present were sufficient to refill the niche with time through local proliferation. Proliferation of *Zeb2*^+/−^ macs also contributed to mac maintenance in the liver. The dual mechanism of replacement in the liver via both BM precursors and local proliferation is consistent with our previous study whereby partial depletion of KCs using the *Clec4f*-*Dtr* mice led to reconstitution of the mac pool via the same two mechanisms (Scott et al., 2016b). This highlights the crucial requirement for ZEB2 within the mac lineage as *Zeb2*^−/−^ macs are outcompeted from the mac niche, regardless of the repopulation mechanism.

How does ZEB2 function to maintain the mac lineage? We propose that loss of ZEB2 leads to loss of the mac tissue-specific identities. In KCs, loss of *Zeb2* leads to loss of *Nr1h3*, suggesting that one mechanism of action of ZEB2 is to maintain the expression of TFs driving the tissue-specific identities of the different mac populations. We found that loss of LXRα in KCs recapitulated the main traits of *Zeb2*^*−/−*^ KCs including loss of KC identity and disappearance from the liver, suggesting that downregulation of LXRα is at least in part responsible for the phenotype of *Zeb2*^*−/−*^ KCs. The mechanisms underlying the control of tissue-specific identities by *Zeb2* in other organs remain to be investigated. However, the reduced expression of the TF *Cebpb* in the *Zeb2*^*−/−*^ AMs, a TF recently reported to be essential for AMs (Cain et al., 2013), suggests that loss of *Zeb2* might control mac identity globally by regulating expression of tissue-specific TFs in the different macs, and we are currently investigating this.

In conclusion, our study highlights that *Zeb2* expression is a defining characteristic of the mac lineage. ZEB2 is crucial for the maintenance of macs, with its absence leading to changes in their transcriptional profiles, including loss of roughly 60% of their tissue-specific identities potentially through the decreased expression of tissue-specific TFs, such as LXRα in KCs. This loss of identity inevitably results in mac disappearance, possibly due to death by necroptosis, identifying ZEB2 as a crucial TF in mac biology and LXRα as a master TF in KCs.

## STAR★Methods

### Key Resources Table

**Table:** 

REAGENT or RESOURCE	SOURCE	IDENTIFIER
**Antibodies**		

CD64 – BV711	Biolegend	139311; RRID: AB_2563846
F4/80 - Biotin	eBioscience	13-4801-85; RRID: AB_466658
CD11c – PE-eFluor 610	eBioscience	61-0114-82; RRID: AB_2574530
CD11b – PE-Cy7	BD Biosciences	552850; RRID: AB_394491
CD11b – Horizon V450	BD Biosciences	560455; RRID: AB_1645266
Clec4F - Unconjugated	R & D Systems	AF2784; RRID: AB_2081339
SiglecF - PE	BD Biosciences	552126; RRID: AB_394341
SiglecF – BUV395	BD Biosciences	740280
SiglecF – Unconjugated	BD Biosciences	552125; RRID: AB_394340
Ly6G - FITC	BD Biosciences	551460; RRID: AB_394207
Ly6G - PE	BD Biosciences	551461; RRID: AB_394208
Ly6G - APC	BD Biosciences	560599; RRID: AB_1727560
CD26 - FITC	BD Biosciences	559652; RRID: AB_397295
CD45 – BV510	Biolegend	103138; RRID: AB_2563061
MHCII – AF700	eBioscience	56-5321-82; RRID: AB_494009
Tim4 – PerCP-eFluor710	eBioscience	46-5866-82; RRID: AB_2573781
Tim4 – AF647	Biolegend	130008; RRID: AB_2271648
Tim4 - PE	eBioscience	12-5866-82; RRID: AB_1257163
Ly6C – eFluor450	eBioscience	48-5932-82; RRID: AB_10805519
Ly6C - FITC	BD Biosciences	553104; RRID: AB_394628
CD20 - PE	eBioscience	12-0203-82; RRID: AB_2572552
CD101 - PE	eBioscience	12-1011-82; RRID: AB_1210728
CD326 (EpCam) – PE-Cy7	Biolegend	118216; RRID: AB_1236471
RFP – Unconjugated	Rockland	600-401-379; RRID: AB_2209751
F4/80 - Unconjugated	ABD Pharmingen Serotec	MCA497R; RRID: AB_323279
CD55 – PE	Biolegend	131803; RRID: AB_1279267
F4/80 – BV786	Biolegend	123141; RRID: AB_2563667

**Chemicals, Peptides, and Recombinant Proteins**		

BV605 – Streptavidin	BD Biosciences	563260
Donkey Anti-Goat IgG – AF647	Thermo Fisher	A-21447; RRID: AB_2535864
Donkey Anti-Goat IgG – AF488	Thermo Fisher	A-11055; RRID: AB_2534102
DAPI	Invitrogen	D1306; RRID: AB_2629482
Donkey Anti-Rat Cy3	Jackson Immunoresearch	712-166-153; RRID: AB_2340669
Donkey Anti-Rabbit - AF647	Invitrogen	A21247; RRID: AB_141778
Tamoxifen	Sigma-Aldrich	T5648-1G
Corn Oil	Sigma-Aldrich	C8267-500ML
Sensifast SYBR no Rox mix	Bioline	BIO-98020
Saponin	Sigma-Aldrich	4521
Donkey Serum	Abcam	ab7475
Rat Serum	Sigma Aldrich	R9759
FoxP3 Transcription factor staining buffer kit	eBioscience	00-5523-00

**Critical Commercial Assays**		

PrimeFlow RNA Assay	Thermo Fisher	88-18005-210
*Zeb2* probes Type 1 for PrimeFlow	Thermo Fisher	PF-210
RNeasy Micro Plus kit	QIAGEN	74034
Sensifast cDNA synthesis kit	Bioline	BIO-65054
Allin Red Taq	HighQu	HQ.HSM0305
CSF1fc	David Hume/Clare Pridans	(Gow et al., 2014)

**Deposited Data**		

RAW and analyzed data	GEO	GSE117081

**Experimental Models: Organisms/Strains**		

Mouse: *Zeb2*^fl/fl^	Huylebroeck Lab	(Higashi et al., 2002)
Mouse: *Clec4f-cre*	CIPHE, Marseille, France	This study
Mouse: *Itgax-cre*	Jackson Laboratories	Stock No: 008068
Mouse: Rosa-RFP	Malissen Lab	(Luche et al., 2007)
Mouse: *Rosa-26-cre*^ert2^	Jackson Laboratories	Stock No: 008463
Mouse CD45.1	Harlan	Stock: B6.SJL-PtprcaPep3b/BoyJ
Mouse *Fcgr1-cre*	Bernard Malissen, Marseille, France	This study
Mouse *Nr1h3*^fl/fl^	Institut de la Souris, GIE, France	(A-Gonzalez et al., 2013, Zhang et al., 2012)

**Oligonucleotides**		

β-Actin qPCR FWD:		GCTTCTAGGCGGACTGTTACTGA
β-Actin qPCR REV:		GCCATGCCAATGTTGTCTCTTAT
*Zeb2* qPCR FWD:		CCAGAGGAAACAAGGATTTCAG
*Zeb2* qPCR REV:		AGGCCTGACATGTAGTCTTGTG
*Zeb2* common (Flox or deletion) PCR		GGGGTCTCCACAGAGTTGAT
*Zeb2* deletion PCR		TGTTTGTTTTGGAGACCGGA
*Zeb2* flox PCR		CTTGCAGTTTGGGCATTCGT
*Nr1h3* qPCR FWD		CAAGGGAGCACGCTATGTCTG
*Nr1h3* qPCR REV		GGACACCGAAGTGGCTTGAG
*Cd5l* qPCR FWD		GAGGACACATGGATGGAATGT
*Cd5l* qPCR REV		ACCCTTGTGTAGCACCTCCA
*Apoc1* qPCR FWD		TCCTGTCCTGATTGTGGTCGT
*Apoc1* qPCR REV		CCAAAGTGTTCCCAAACTCCTT
*Cdh5* qPCR FWD		CACTGCTTTGGGAGCCTTC
*Cdh5* qPCR REV		GGGGCAGCGATTCATTTTTC

**Software and Algorithms**		

GraphPad Prism 6	GraphPad Software, Inc., California	
FlowJo 10.2	TreeStar, FlowJo LLC, Ashland, Oregon	
Image J	NIH, Bethesda, Maryland	

### Contact for Reagent and Resource Sharing

Further information and requests for resources and reagents should be directed to and will be fulfilled by the lead contact, Martin Guilliams (martin.guilliams@irc.vib-ugent.be).

### Experimental Model and Subject Details

#### In Vivo Animal Studies

The following mice were used in this study; *Zeb2*^fl/fl^ (Higashi et al., 2002), *Nr1h3*^fl/fl^ *Itgax-cre* (Caton et al., 2007), Rosa-RFP (Luche et al., 2007), *Rosa-26-cre*^ert2^ (Ventura et al., 2007), *Clec4f-cre* (B6-*Clec4f* ^tm3Ciphe^; were developed by the Centre d’Immunophenomique, Marseille, France) and *Fcgr1-cre* (B6-*Fcgr1*^tm3Ciphe^; generated by Bernard Malissen). All mice were used on a C57Bl/6 background and a mix of male and female mice were used for each experiment. Mice were used between 6 and 8 weeks of age unless otherwise stated. All mice were bred and maintained at the VIB (Ghent University) under specific pathogen free conditions. All animals were randomly allocated to experimental groups and littermate controls were used in all experiments. All experiments were performed in accordance with the ethical committee of the Faculty of Science of the VIB.

#### Construction of Clec4f-IRES-iCRE Mice

Using ET recombination, an IRES-iCRE-loxP-Cre-NeoR-loxP cassette was introduced in the 3’ untranslated region of the *Clec4f* gene, downstream of the stop codon. JM8.F6 C57BL/6N ES cells (Pettitt et al., 2009) were electroporated with the targeting vector. After selection in G418, ES cell clones were screened for proper homologous recombination by Southern blot. A neomycin-specific probe was used to ensure that adventitious non-homologous recombination events had not occurred in the selected ES clones. Properly recombined ES cells were injected into FVB blastocysts. Germline transmission led to the self-excision of the loxP-Cre-NeoR-loxP cassette in male germinal cells. The resulting *Clec4f-IRES-iCRE* allele (official name B6-*Clec4f*^m2Ciphe^, called here *Clec4f-cre)* was identified by PCR of tail DNA. The primers: sense 5'-GATTCCCCTTCAGACCCTGAAT-3’, sense 5’-TGATGAACTACATCAGAACCTGG-3’ and antisense 5’-TATTGAGGGCTTATCTGGGC-3’ amplify a 496 bp band in case of the wild-type *Clec4f* allele and a 304 bp band in the case of the *Clec4f-IRES-iCre* allele.

#### Construction of Fcgr1-IRES-iCRE-2A-TEAL Mice

Using ET recombination, an IRES-iCRE-2A-TEAL-frt-neoR-frt cassette was introduced in the 3’ untranslated region of the *Fcgr1* gene, downstream of the stop codon. The targeting construct was abutted to a cassette coding for the diphtheria toxin fragment A, and linearized with Pme1. JM8.F6 C57BL/6N ES cells (Pettitt et al., 2009) were electroporated with the targeting vector. After selection in G418, ES cell clones were screened for proper homologous recombination by Southern blot. A neomycin-specific probe was used to ensure that adventitious non-homologous recombination events had not occurred in the selected ES clones. Properly recombined ES cells were injected into FVB blastocysts. Upon germline transmission, mice were then crossed to mice expressing the site-specific recombinase FLP (Kranz et al., 2010) to delete the frt-flanked neoR cassette. The resulting *Fcgr1-IRES-iCRE-TEAL* floxed allele (official name B6-*Fcgr1*^tm2Ciphe^, called here *Fcgr1-cre)* was identified by PCR of tail DNA. The primers: sense 5'-CCCTTCCTCCCAGTGACAGTACTG-3', sense 5’-GACGGCATGGACGAGCTGTACA-3’ and antisense 5'-TGAACCCATCCACCCTGTGAG-3' amplify a 402 bp band in case of the wild-type *Fcgr1* allele and a 464 bp band in the case of the *Fcgr1-IRES-iCre-TEAL* allele.

### Method Details

#### Isolation of Tissue Leukocytes

For the isolation of liver leukocytes, livers were isolated from PBS-perfused mice, chopped finely and subjected to GentleMACS dissociation and incubated for 20 min with 1 mg/ml Collagenase A (Sigma) and 10U/ml DNase (Roche) in a shaking water bath at 37°C. Following a second round of GentleMACS dissociation, single cell suspensions were filtered over a 100um filter. For the isolation of lung, brain and spleen leukocytes, lungs, brains, kidneys and spleens were isolated from PBS-perfused mice finely chopped and incubated for 30 min with 0.2 mg/ml Liberase TM (Roche) and 10 U/ml DNase (Roche) in a shaking water bath at 37°C. Single cell suspensions from brain were then subjected to a 100:40 percoll gradient (Sigma) to isolate leukocytes. Colonic intestinal lamina propria leukocytes were isolated as described previously (Bain et al., 2013, Scott et al., 2015).

#### Generation of BM Chimeras

Partially-protected: 6 week-old *Clec4f-cre*x*Zeb2*^fl/fl^, *Itgax-cre*X*Zeb2*^fl/fl^ or *Zeb2*^fl/fl^ littermate controls (CD45.2) were anaesthetized by intraperitoneal administration of Ketamine (150 mg/kg) and Xylazine (10 mg/kg). Livers or lungs were protected with a 3-cm-thick lead cover before mice were lethally irradiated with 8 Gy. Once recovered from the anesthesia, mice were reconstituted by intravenous administration of 10x10^6^ BM cells from congenic CD45.1 or CD45.1/CD45.2 BM from wild-type mice. 4 weeks after irradiation chimerism in the blood and liver or lung was assessed by flow cytometry.

Non-protected: 6-8 week old CD45.1 or CD45.1xCD45.2 WT mice were lethally irradiated with 8 Gy. Mice were reconstituted with 2-3X10^6^ BM cells from gender-matched *Zeb2*^fl/fl^ or *Rosa-26-cre*^*ert2*^x*Zeb2*^fl/fl^ (CD45.2) mice. At least 8 weeks post irradiation mice were fed 5mg tamoxifen by oral gavage daily for 5 days before being sacrificed at the indicated time-points after the final dose.

#### Flow Cytometry

Cells (0.5–5 106) were stained with appropriate antibodies at 4°C in the dark for 30-45 mins and were analyzed with a Fortessa (BD Biosciences) and FlowJo software (TreeStar). KCs, AMs, splenic macs, colonic macs and microglia were sorted using an ARIA II or ARIA III (BD, Biosciences). The full list of antibodies used can be found in the Key Resource Table. Primeflow assay (Thermo Fisher) for *Zeb2* expression was performed in 96-well U bottom plates according to the manufacturer’s instructions using commercially available *Zeb2* primers (Thermo Fisher).

#### Microarray

25000 AMs and Microglia from WT mice were sorted into 500ul RLT buffer (QIAGEN). RNA was isolated using the RNeasy micro kit (QIAGEN) and sent to the Nucleomics facility, VIB Leuven, Belgium where the microarrays were performed using the GeneChip Mouse Gene 1.0 ST arrays (Affymetrix). Samples were subsequently analyzed using R/Bioconductor. All samples passed quality control, and the Robust Multi-array Average (RMA) procedure was used to normalize data within arrays (probeset summarization, background correction and log2-transformation) and between arrays (quantile normalization). Only probesets that mapped uniquely to one gene were kept, and for each gene, the probeset with the highest expression level was kept.

#### Bulk RNA Sequencing

25,000 KCs or AMs were FACS-purified into 500μl of RLT plus buffer (QIAGEN) and β-mercaptoethanol. RNA was isolated using a RNeasy Plus micro kit (QIAGEN) and sent to the VIB Nucleomics facility, where the RNA sequencing was performed using a NextSeq sequencer (Illumina). The pre-processing of the RNA sequencing data was done by Trimmomatic. The adapters were cut and reads were trimmed when the quality dropped below 20. Reads with a length <35 were discarded. All samples passed quality control based on the results of FastQC. Reads were mapped to the mouse reference genome via Tophat2 and counted via HTSeqCount. Samples were subsequently analyzed using R/Bioconductor, and the limma (voom) procedure was used to normalize the data.

#### Single Cell RNA Sequencing

##### Sorting and RNA Isolation

60000 Clec4F^+^CD64^+^F4/80^+^CD45^+^ Live cells from livers of *Clec4-cre*x*Zeb2*^fl/fl^ and *Zeb2*^fl/fl^ littermate controls, 60000 CD11c^+^SiglecF^+^F4/80^+^CD64^+^CD45^+^ Live cells from lungs of *Itgax-cre*x*Zeb2*^fl/fl^ and *Zeb2*^fl/fl^ littermate controls, 20000 CD45^int^, F4/80^+^CD64^+^ live cells from brains of *Fcgr1-cre*x*Zeb2*^fl/fl^ and *Zeb2*^fl/fl^ littermate controls, 60000 CD45^+^Ly6C^-^Ly6G^-^SiglecF^-^CD64^+^F4/80^+^ live cells from colons of *Fcgr1-cre*x*Zeb2*^fl/fl^ and *Zeb2*^fl/fl^ littermate controls and 60000 CD45^+^Ly6C^-^Ly6G^-^SiglecF^-^CD64^+^F4/80^+^ live cells from spleens of *Fcgr1-cre*x*Zeb2*^fl/fl^ and *Zeb2*^fl/fl^ littermate controls were FACS-purified. Cells were sorted into PBS 0.04% BSA, spun down and resuspended in PBS with 0.04%BSA at an estimated final concentration of 1000 cells/μl. Cellular suspensions (target recovery of 6000 cells) were loaded on a GemCode Single-Cell Instrument (10x Genomics, Pleasanton, CA, USA) to generate single-cell Gel Bead-in-EMulsion (GEMs). Single-cell RNA-Seq libraries were prepared using GemCode Single-Cell 3ʹGel Bead and Library Kit (10x Genomics) according to the manufacturer’s instructions. Briefly, GEM-RT was performed in 96-deep well reaction module: 55°C for 45min, 85°C for 5 min; end at 4°C. After RT, GEMs were broken down and the cDNA was cleaned up with DynaBeads MyOne Silane Beads (Thermo Fisher Scientific, 37002D) and SPRIselect Reagent Kit (SPRI; Beckman Coulter; B23318). cDNA was amplified with 96-Deep Well Reaction Module: 98°C for 3 min; cycled 12 times : 98°C for 15s, 67°C for 20 s, and 72°C for 1 min; 72°C for 1 min; end at 4°C. Amplified cDNA product was cleaned up with SPRIselect Reagent Kit prior to enzymatic fragmentation. Indexed sequencing libraries were generated using the reagents in the GemCode Single-Cell 3ʹ Library Kit with the following intermediates: (1) end repair; (2) A-tailing; (3) adapter ligation; (4) post-ligation SPRIselect cleanup and (5) sample index PCR. Pre-fragmentation and post-sample index PCR samples were analysed using the Agilent 2100 Bioanalyzer.

##### RNA Sequencing Analysis

Sequencing libraries were loaded on an Illumina NextSeq500 (KCs, AMs) or HiSeq (Microglia, splenic macs, colonic macs) with sequencing settings following recommendations of 10X Genomics (26/8/0/98 - 2.1pM loading concentration). Sequencing was performed at the VIB Nucleomics Core (VIB, Leuven). The demultiplexing of the raw data was done by the 10x’s CellRanger software (version 2.0.0 (KCs, AMs) or version 2.0.2 (Microglia, splenic macs, colonic macs); cellranger mkfastq which wraps Illumina's bcl2fastq). The reads obtained from the demultiplexing were used as the input for ‘cellranger count’ (10x’s CellRanger software) which align the reads to the mouse reference genome (mm10) using STAR and collapses to unique molecular identifier (UMI) counts. The result is a large digital expression matrix with cell barcodes as rows and gene identities as columns. The aggregation of the CRE- and CRE+ samples was done using ‘cellranger aggr’ (10x’s CellRanger software). For heatmaps, each column corresponds to a single cell.

##### Preprocessing Data

Preprocessing of the data was done by the scran and scater R package according to workflow proposed by the Marioni lab (Lun et al., 2016). Outlier cells were identified based on 3 metrics (library size, number of expressed genes and mitochondrial proportion) and cells were tagged as outliers when they were 3 median absolute deviation (MADs) away from the median value of each metric across all cells. Low-abundance genes were removed using the ‘calcAverage’ function and the proposed workflow. The raw counts were normalised and log2 transformed by first calculating “size factors” that represent the extent to which counts should be scaled in each library. Detecting highly variable genes, finding clusters and creating tSNE plots was done using the Seurat pipeline. Marker genes per identified subpopulation were found using the findMarker function of the Seurat pipeline. Additional low quality (low UMI counts, high % of mitochondrial genes), contaminating (potential doublets) and actively proliferating cells were also removed from the analysis (Figure S1G).

#### Gene-Expression Analysis by Real-Time RT-PCR

RNA was purified from 10000-25000 sorted cells using an RNeasy Plus micro kit (QIAGEN). RNA was reverse transcribed to cDNA with an iScript Advanced cDNA Synthesis kit (Bio-Rad Laboratories). Gene expression was assayed by real-time RT-PCR using a SensiFast SYBR NoRox kit (GC Biotech) on a PCR amplification and detection instrument (LightCycler 480; Roche) with the primers listed in the Key Resource Table. Gene expression was normalized to β-actin, and the mean relative gene expression was calculated using the 2−ΔΔC(t) method.

#### Confocal Microscopy

2-3mm slices of livers were fixed by immersion in Antigen fix (Diapath) for 1h, washed in PBS, infused overnight in 30% sucrose and frozen in Tissue-Tek OCT compound (Sakura Finetek) for cryostat sectioning. After permeabilization with 0.5% saponin and unspecific binding site blocking with 2% bovine serum albumin, 1% fetal calf serum and 1% donkey serum for 30 minutes, 14μm–thick cryostat tissue sections were labeled overnight at 4°C with primary antibodies followed by incubation for 1 hour at room temperature with secondary antibodies. When two rat antibodies were used on the same section, the directly conjugated rat antibody was incubated for 1h after blocking with a donkey anti-rat secondary antibody with 1% rat serum for 30 minutes. Slices were mounted on ProLong Diamond (Thermo Fisher Scientific) and imaged with a Zeiss LSM 780 confocal microscope (Carl Zeiss, Oberkochen, Germany). Images were analyzed using ImageJ software.

#### PCR Analysis of Zeb2 Deletion

25000 cells of required phenotype and genotype were FACS-purified from livers and lungs of *Clec4f-cre*x*Zeb2*^fl/fl^ and *Itgax-cre*x*Zeb2*^fl/fl^ mice respectively. DNA was extracted by boiling the cells at 95C in 50μl 50mM NaOH for 20minutes. After boiling 5μl 1.5M Tris pH8.8 was added to the cells. 1 or 0.2μl pf extracted DNA was added to a PCR reaction containing primer pairs (*Zeb2* PCR) listed in the key resource table and Allin Red Taq polymerase (HighQu). PCR protocol was as follows: 95C 1min, 40 cycles of 95C 15secs, 62C 15secs, 72C 30secs and a 5min incubation at 72C. PCR products were resolved on a 2% agarose gel.

### Quantification and Statistical Analysis

In all experiments, data are presented as mean ± SEM unless stated otherwise. Statistical tests were selected based on appropriate assumptions with respect to data distribution and variance characteristics. Student’s t test (two-tailed) was used for the statistical analysis of differences between two groups. One-way ANOVA with Bonferroni post-test was used for the statistical analysis of differences between more than two groups. Statistical significance was defined as p < 0.05. Sample sizes were chosen according to standard guidelines. Number of animals is indicated as ‘‘n.’’ Of note, sizes of the tested animal groups were also dictated by availability of the transgenic strains and litter sizes, allowing littermate controls. Pre-established exclusion criteria are based on IACUC guidelines. The investigator was not blinded to the mouse group allocation.

### Data and Software Availability

All RNA-sequencing data have been deposited in the Gene Expression Omnibus public database under accession number GSE117081.

## Supplemental Information

Document S1. Figures S1–S7

Table S1. DE Genes across Groups from KCs Sorted from *Clec4f-Cre*x*Zeb2*^fl/fl^ Mice, Related to Figure 1

Table S2. DE Genes across Groups from AMs Sorted from *Itgax-Cre*x*Zeb2*^fl/fl^ Mice, Related to Figure 1

Table S3. DE Genes across Groups from KCs Sorted from *Clec4f-cre*x*Nr1h3*^fl/fl^ Mice, Related to Figure 4

Table S4. DE Genes across Groups from Splenic Macs Sorted from *Fcgr1-cre*x*Zeb2*^fl/fl^ Mice, Related to Figures 6 and 7

Table S5. DE Genes across Groups from Microglia Sorted from *Fcgr1-cre*x*Zeb2*^fl/fl^ Mice, Related to Figures 6 and 7

Table S6. DE Genes across Groups from Colonic Macs Sorted from *Fcgr1-cre*x*Zeb2*^fl/fl^ Mice, Related to Figures 6 and 7

Document S2. Article plus Supplemental Information
