# Supplementary material for: The Transcription Factor ZEB2 Is Required to Maintain the Tissue-Specific Identities of Macrophages
Source: Immunity. 2018 Aug 21;49(2):312–325.e5. doi: 10.1016/j.immuni.2018.07.004 (PMC6104815; doi:10.1016/j.immuni.2018.07.004)
Supplement: Document S1. Figures S1–S7 [file mmc1.pdf]

Immunity, Volume *49*

## Supplemental Information

# The Transcription Factor ZEB2 Is Required to Maintain the Tissue-Specific Identities of Macrophages

**Charlotte L. Scott, Wouter T'Jonck, Liesbet Martens, Helena Todorov, Dorine Sichien, Bieke Soen, Johnny Bonnardel, Sofie De Prijck, Niels Vandamme, Robrecht Cannoodt, Wouter Saelens, Bavo Vanneste, Wendy Toussaint, Pieter De Bleser, Nozomi Takahashi, Peter Vandenabeele, Sandrine Henri, Clare Pridans, David A. Hume, Bart N. Lambrecht, Patrick De Baetselier, Simon W.F. Milling, Jo A. Van Ginderachter, Bernard Malissen, Geert Berx, Alain Beschin, Yvan Saeys, and Martin Guilliams**

# SUPPLEMENTARY INFORMATION

**The transcription factor Zeb2 is required to maintain the tissue-specific identities of macrophages**

**Scott et al.**

**Figures S1-S7**

**Tables S1-S8**

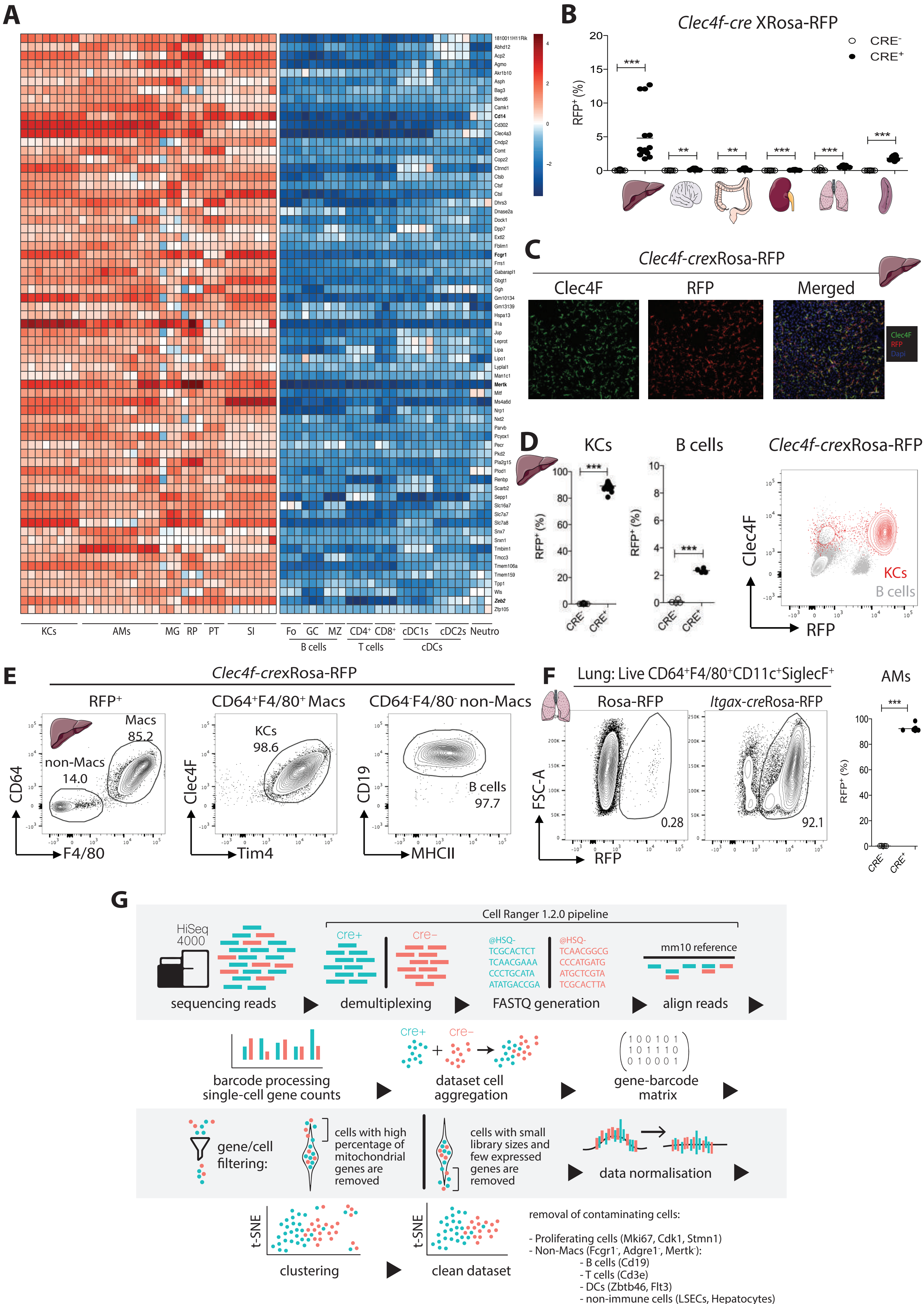

A
1810011H11Rik
Abhd12
Acp2
Agmo
Akr1b10
Asph
Bag3
Bend6
Camk1
Cd14
Cd302
Clec4a3
Cndp2
Comt
Copz2
Ctnnd1
Ctsb
Ctsf
Ctsl
Dhrs3
Dnase2a
Dock1
Dpp7
Extl2
Fblim1
Fcgr1
Frrs1
Gabarapl1
Gbgt1
Ggh
Gm10134
Gm13139
Hspa13
Il1a
Jup
Leprot
Lipa
Lipo1
Lypla1
Man1c1
Mertk
Mitf
Ms4a6d
Nrp1
Nxt2
Parvb
Pcyox1
Pecr
Pkd2
Pla2g15
Plod1
Renbp
Scarb2
Sepp1
Slc16a7
Slc7a7
Slc7a8
Snx7
Srxn1
Tmbim1
Tmcc3
Tmem106a
Tmem159
Tpp1
Wls
Zeb2
Zfp105
KCs
AMs
MG
RP
PT
SI
Fo
GC
MZ
B cells
CD4+
CD8+
T cells
cDC1s
cDC2s
cDCs
Neutro
B
Clec4f-cre XRosa-RFP
CRE-
CRE+
RFP+ (%)
C
Clec4f-crexRosa-RFP
Clec4F
RFP
Merged
Clec4F
RFP
Dapi
D
KCs
B cells
Clec4f-crexRosa-RFP
RFP+ (%)
CRE-
CRE+
Clec4F
RFP
KCs
B cells
E
Clec4f-crexRosa-RFP
RFP+
CD64+F4/80+ Macs
CD64-F4/80- non-Macs
Macs
85.2
non-Macs
14.0
CD64
F4/80
KCs
98.6
Clec4F
Tim4
B cells
97.7
CD19
MHCII
F
Lung: Live CD64+F4/80+CD11c+SiglecF+
Rosa-RFP
Itgax-creRosa-RFP
AMs
FSC-A
RFP
0.28
92.1
RFP+ (%)
CRE-
CRE+
G
Cell Ranger 1.2.0 pipeline
HiSeq
4000
sequencing reads
cre+
cre−
demultiplexing
@HSQ-
TCGCACTCT
TCAACGAAA
CCCTGCATA
ATATGACCGA
@HSQ-
TCAACGGCG
CCCATGATG
ATGCTCGTA
TCGCACTTA
FASTQ generation
mm10 reference
align reads
barcode processing
single-cell gene counts
cre+
cre−
dataset cell
aggregation
1 0 0 1 0 1
1 0 1 1 1 0
0 1 0 0 0 1
gene-barcode
matrix
gene/cell
filtering:
cells with high
percentage of
mitochondrial
genes are
removed
cells with small
library sizes and
few expressed
genes are
removed
data normalisation
t-SNE
clustering
t-SNE
clean dataset
removal of contaminating cells:
- Proliferating cells (Mki67, Cdk1, Stmn1)
- Non-Macs (Fcgr1-, Adgre1-, Mertk-):
- B cells (Cd19)
- T cells (Cd3e)
- DCs (Zbtb46, Flt3)
- non-immune cells (LSECs, Hepatocytes)

**Figure S1; Related to Figure 1: *Zeb2* expression, validation of CRE lines and SC-RNA-Seq analysis pipeline.**

(A) Heatmap showing relative expression of genes (normalized per mean expression of each gene) identified to be shared among tissue resident macs and lacking from other immune cells including splenic B cells, naïve T cells, cDC1s, cDC2s and blood neutrophils. Data are from microarray analyses performed either in house or by the Immgen consortium. (B) % of RFP-expressing cells in indicated tissues of *Clec4f-cre*xRosa-RFP$^{+/-}$ (CRE$^{+}$) and Rosa-RFP$^{+/-}$ (CRE$^{-}$) mice. Data are pooled from 2-3 experiments with n=8-16 per group. **p<0.01, ***p<0.001 Student's t test. (C) Expression of Clec4F, RFP and DAPI by confocal microscopy in liver of *Clec4f-cre*xRosa-RFP$^{+/-}$ mice. Data are representative of 2 experiments with n=9. (D) % of KCs (Clec4F$^{+}$Tim4$^{+}$CD64$^{+}$F4/80$^{+}$) and B cells (CD19$^{+}$MHCII$^{+}$) expressing RFP in *Clec4f-cre*xRosa-RFP$^{+/-}$ mice (CRE$^{+}$) and Rosa-RFP$^{+/-}$ mice (CRE$^{-}$) and expression of Clec4F and RFP by KCs (red) and B cells (grey) in the liver of *Clec4f-cre*xRosa-RFP$^{+/-}$ mice. Data are pooled from 3 (KCs) or 1(B cells) experiment(s) with n=4-16 per group. ***p<0.001 Student's t test. (E) Expression of CD64, F4/80, Clec4F, Tim4, CD19 and MHCII on RFP$^{+}$ cells in the liver of *Clec4f-cre*xRosa-RFP$^{+/-}$ mice. Data are representative of 3 experiments with n=12-16 per group. (F) Expression of RFP by CD11c$^{+}$CD64$^{+}$F4/80$^{+}$SiglecF$^{+}$ AMs and % of AMs expressing RFP in *Itgax-cre*xRosaRFP$^{+/-}$ (CRE$^{+}$) and Rosa-RFP$^{+/-}$ (CRE$^{-}$) mice. Data are pooled from 2 experiments with n=5-9 per group. ***p<0.001 Student's t test. (G) Pipeline followed for analysis of SC-RNA-Seq data.

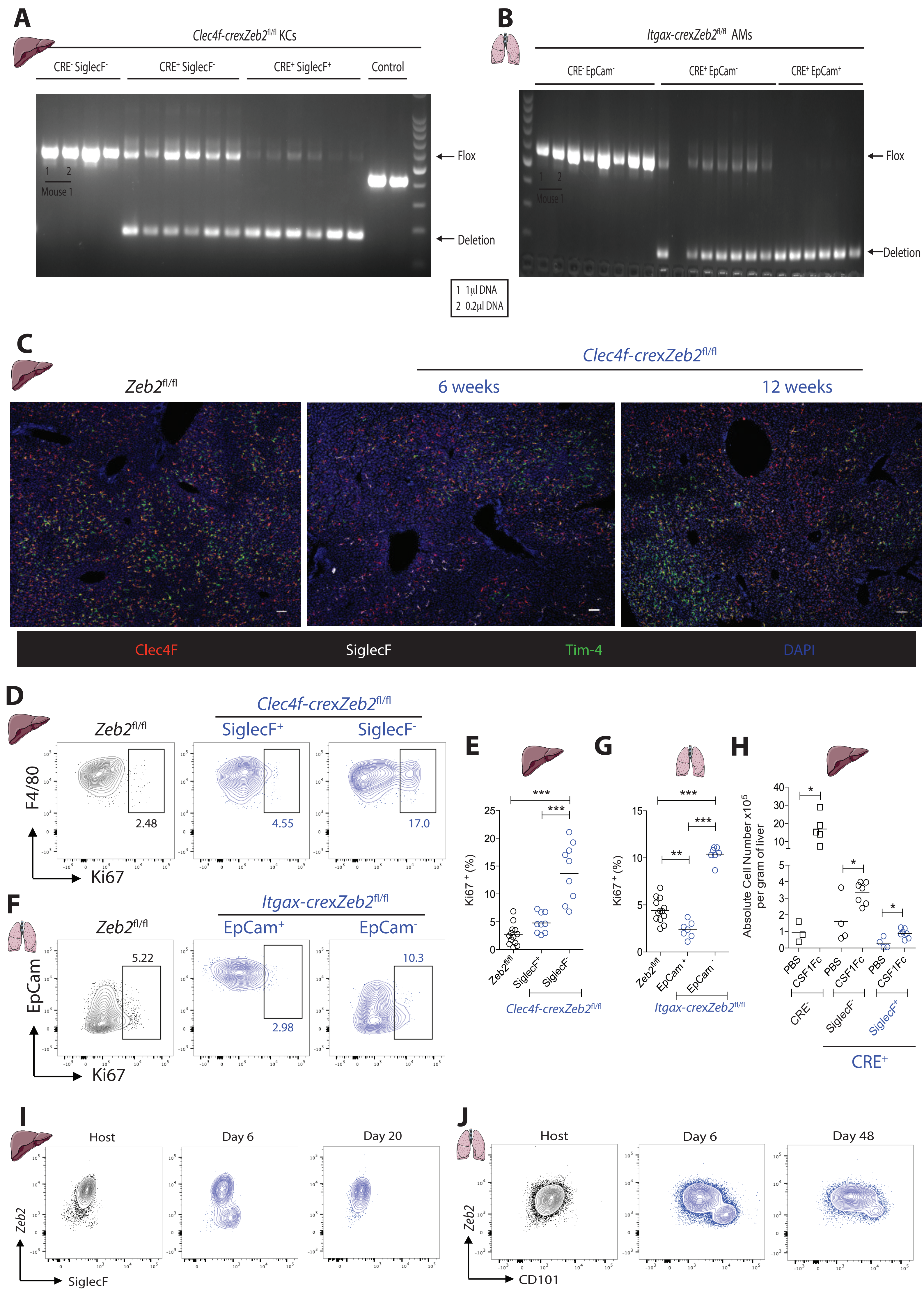

A
Clec4f-crexZeb2fl/fl KCs
CRE- SiglecF-
CRE+ SiglecF-
CRE+ SiglecF+
Control
Flox
Deletion
Mouse 1
1 1µl DNA
2 0.2µl DNA
B
Itgax-crexZeb2fl/fl AMs
CRE- EpCam-
CRE+ EpCam-
CRE+ EpCam+
C
Zeb2fl/fl
Clec4f-crexZeb2fl/fl
6 weeks
12 weeks
Clec4F
SiglecF
Tim-4
DAPI
D
F4/80
Ki67
SiglecF+
SiglecF-
2.48
4.55
17.0
E
Ki67+ (%)
F
EpCam
Itgax-crexZeb2fl/fl
EpCam+
EpCam-
5.22
2.98
10.3
G
H
Absolute Cell Number x10^5 per gram of liver
PBS
CSF1Fc
CRE-
CRE+
I
Host
Day 6
Day 20
Zeb2
SiglecF
J
Day 48
CD101

**Figure S2; Related to Figure 2: Presence of *Zeb2*$^{+/-}$ macs in liver and lung of conditional KO mice**

(A,B) 2% Agarose gel showing results of genomic PCR for *Zeb2* flox and *Zeb2* deletion band in indicated mac populations from (A) *Clec4f-cre*x*Zeb2*$^{fl/fl}$ and (B) *Itgax-cre*x*Zeb2*$^{fl/fl}$ mice compared with Zeb2$^{fl/fl}$ littermate controls. All samples are run in duplicate. For the PCR reaction 1μl DNA loaded was in the first well of each sample and 0.2μl DNA was loaded in the second well. (C) Expression of Clec4F, Tim4, SiglecF and DAPI by confocal microscopy in liver of *Clec4f-cre*x*Zeb2*$^{fl/fl}$ mice at 6 and 12 weeks of age compared with *Zeb2*$^{fl/fl}$ livers. Data are representative of 1 experiment with n=2 per group. (D,E) Expression of F4/80 and Ki-67 by indicated KC populations. Data are pooled from 2 experiments with n=8-14 per group. ***p<0.001, One way ANOVA with Bonferroni post-test (F,G) Expression of EpCam and Ki-67 by indicated AM populations. Data are pooled from 2 experiments with n=4-9 per group. ***p<0.001, One way ANOVA with Bonferroni post-test. (H) Absolute number of KCs in *Zeb2*$^{fl/fl}$ (CRE$^{-}$) and *Clec4f-cre*x*Zeb2*$^{fl/fl}$ (CRE$^{+}$) mice which received 1mg/kg CSF1Fc subcutaneously for 4 days or PBS as a control before being sacrificed on day 6. (I) *Zeb2* mRNA expression (PrimeFlow) and SiglecF expression in CD45.2 donor KCs (blue) and CD45.1 host KCs (black) at indicated time-points. (J) *Zeb2* mRNA expression (PrimeFlow) and CD101 expression in CD45.2 donor AMs (blue) and CD45.1 host AMs (black) at indicated time-points.

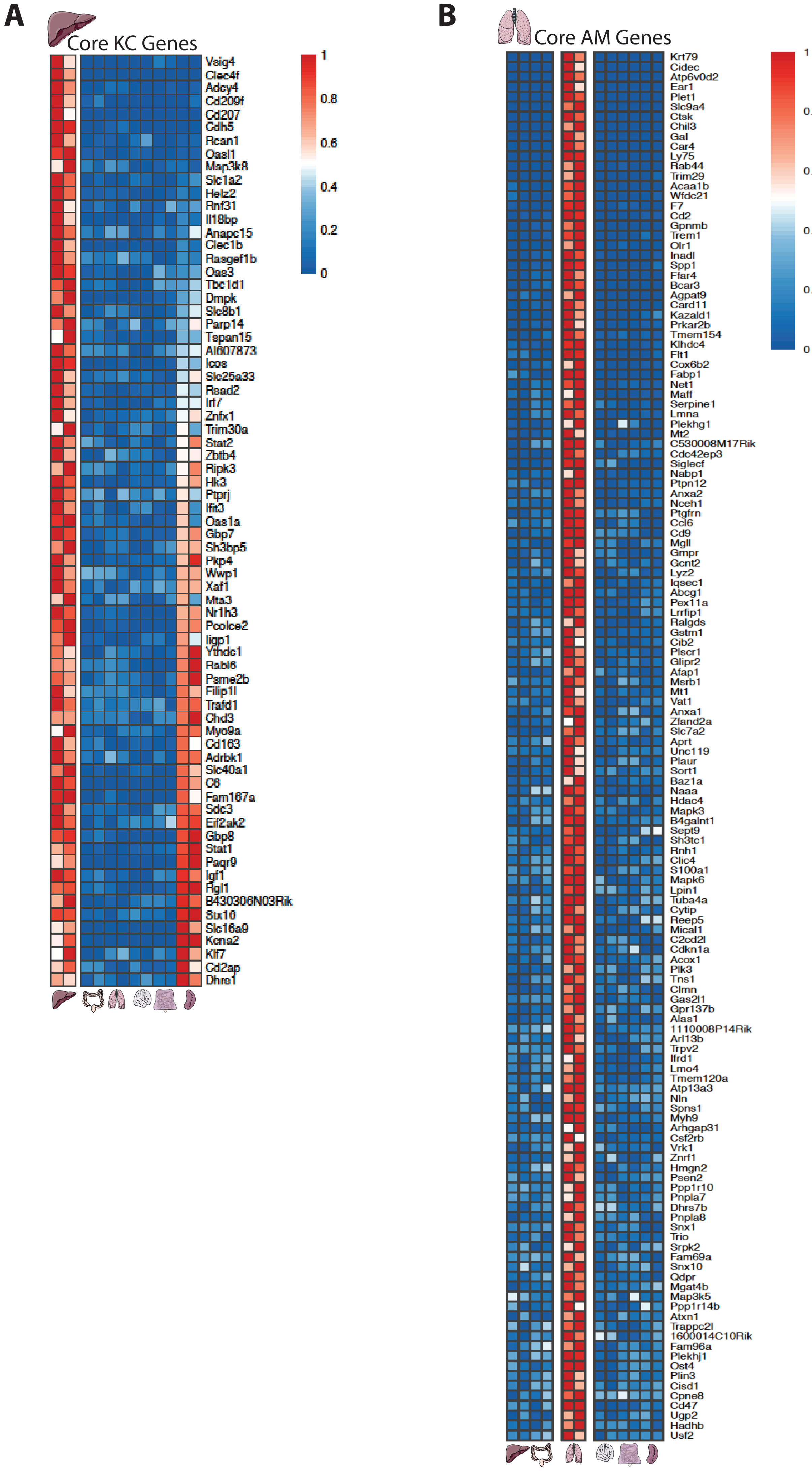
A
Core KC Genes
Vsig4
Clec4f
Adcy4
Cd209f
Cd207
Cdh5
Rcan1
Oasl1
Map3k8
Slc1a2
Helz2
Rnf31
Il18bp
Anapc15
Clec1b
Rasgef1b
Oas3
Tbc1d1
Dmpk
Slc8b1
Parp14
Tspan15
AI607873
Icos
Slc25a33
Rsad2
Irf7
Znfx1
Trim30a
Stat2
Zbtb4
Ripk3
Hk3
Ptprj
Ifit3
Oas1a
Gbp7
Sh3bp5
Pkp4
Wwp1
Xaf1
Mta3
Nr1h3
Pcolce2
Iigp1
Ythdc1
Rabl6
Psme2b
Filip1l
Trafd1
Chd3
Myo9a
Cd163
Adrbk1
Slc40a1
C6
Fam167a
Sdc3
Eif2ak2
Gbp8
Stat1
Paqr9
Igf1
Rgl1
B430306N03Rik
Stx16
Slc16a9
Kcna2
Klf7
Cd2ap
Dhrs1
1
0.8
0.6
0.4
0.2
0
B
Core AM Genes
Krt79
Cidec
Atp6v0d2
Ear1
Plet1
Slc9a4
Ctsk
Chil3
Gal
Car4
Ly75
Rab44
Trim29
Acaa1b
Wfdc21
F7
Cd2
Gpnmb
Trem1
Olr1
Inadl
Spp1
Ffar4
Bcar3
Agpat9
Card11
Kazald1
Prkar2b
Tmem154
Klhdc4
Flt1
Cox6b2
Fabp1
Net1
Maff
Serpine1
Lmna
Plekhg1
Mt2
C530008M17Rik
Cdc42ep3
Siglecf
Nabp1
Ptpn12
Anxa2
Nceh1
Ptgfrn
Ccl6
Cd9
Mgll
Gmpr
Gcnt2
Lyz2
Iqsec1
Abcg1
Pex11a
Lrrfip1
Ralgds
Gstm1
Cib2
Plscr1
Glipr2
Afap1
Msrb1
Mt1
Vat1
Anxa1
Zfand2a
Slc7a2
Aprt
Unc119
Plaur
Sort1
Baz1a
Naaa
Hdac4
Mapk3
B4galnt1
Sept9
Sh3tc1
Rnh1
Clic4
S100a1
Mapk6
Lpin1
Tuba4a
Cytip
Reep5
Mical1
C2cd2l
Cdkn1a
Acox1
Plk3
Tns1
Clmn
Gas2l1
Gpr137b
Alas1
1110008P14Rik
Arl13b
Trpv2
Ifrd1
Lmo4
Tmem120a
Atp13a3
Nln
Spns1
Myh9
Arhgap31
Csf2rb
Vrk1
Znrf1
Hmgn2
Psen2
Ppp1r10
Pnpla7
Dhrs7b
Pnpla8
Snx1
Trio
Srpk2
Fam69a
Snx10
Qdpr
Mgat4b
Map3k5
Ppp1r14b
Atxn1
Trappc2l
1600014C10Rik
Fam96a
Plekhj1
Ost4
Plin3
Cisd1
Cpne8
Cd47
Ugp2
Hadhb
Usf2
1
0.8
0.6
0.4
0.2
0

**Figure S3; related to Figure 3: Core KC and AM transcriptional profiles redefined**

(A,B) Heatmap showing expression of core KC (A) or AM (B) genes by KCs, colon macs (LiMacs), AMs (Lung), Microglia, peritoneal macs and splenic macs. Data was previously published by (Lavin et al., 2014) and downloaded from the NCBI for this analysis.

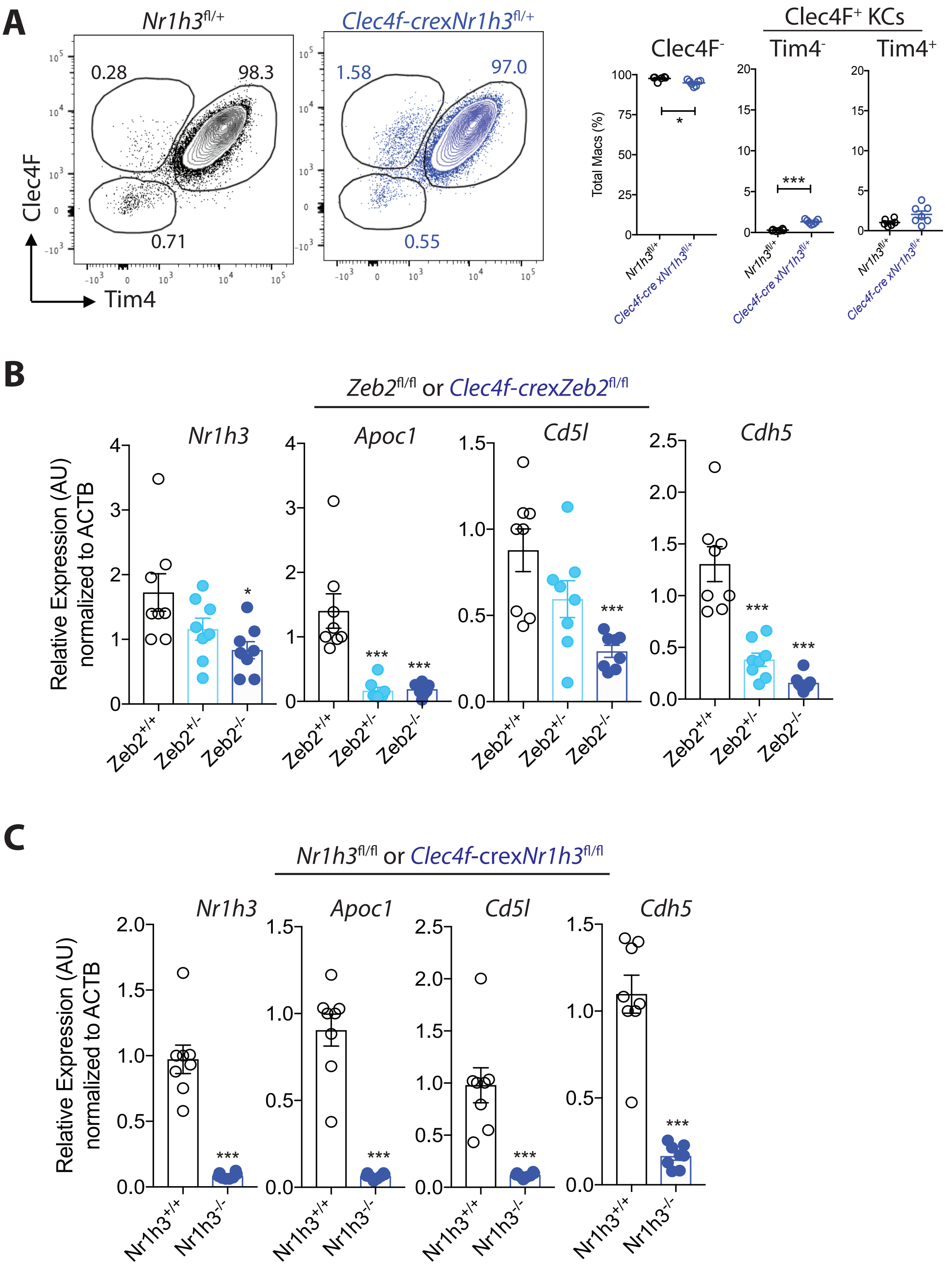


**D**

moKC genes
(DE genes between WT and moKCs D15 Adj P value 0.05, Log FC <1 or >1)

| | | | | |
|---|---|---|---|---|
| Ccr2 | Prelid2 | mt-Tm | Cd163 | Ccdc109b |
| Cx3cr1 | Tceal1 | C6 | Slc27a6 | Cd207 |
| Ccr3 | Hyal4 | C4b | Colec12 | Anpep |
| Tspan32 | Kitl | Kcnj16 | Xlr | Cpne8 |
| Ccr1 | Ecm1 | Fam84a | Sdc2 | Stk39 |
| Ptgs2 | Ryk | Raver2 | Bmpr1a | Apoc1 |
| Lphn3 | Btbd11 | Stard13 | Timd4 | Marco |
| Clec4b1 | Enpp2 | n-R5s89 | Cd209f | Cfh |
| Ifi44 | Klhl13 | 3830403N18 | Cxcl13 | Mcts2 |

**Figure S4; related to Figure 4: Loss of LXRα from KCs mimics main features of loss of ZEB2**

(A) Expression of Clec4F and Tim4 by total liver macs in *Clec4f-cre*x*Nr1h3*$^{fl/+}$ and *Nr1h3*$^{fl/+}$ mice and % of total macs expressing Clec4F and Tim4. Data are pooled from 2 experiments with n=6-7 per group. *p<0.05, ***p<0.001 Student's t test. (B) Relative mRNA expression of indicated genes normalized to β-actin as determined by qPCR of FACS-purified KCs from *Zeb2*$^{fl/fl}$ mice (Zeb2$^{+/+}$), SiglecF$^{-}$ KCs (Zeb2$^{+/-}$) and SiglecF$^{+}$ KCs (Zeb2$^{-/-}$) from *Clec4f-cre*x*Zeb2*$^{fl/fl}$. Data are pooled from 2 experiments with n=8 per group. *p<0.05, ***p<0.001 One way ANOVA with Bonferroni post-test. (C) Relative mRNA expression of indicated genes normalized to β-actin as determined by qPCR of FACS-purified KCs from *Nr1h3*$^{fl/fl}$ mice (Nr1h3$^{+/+}$) or *Clec4f-cre*x*Nr1h3*$^{fl/fl}$ mice (*Nr1h3*$^{-/-}$). Data are pooled from 2 experiments with n=8 per group. ***p<0.001 Student's t-test. (D) Table of DE genes (adj. p value 0.05, log FC <1 or >1) between embryonic KCs and monocyte derived (moKCs) from KC-DTR mice 15 days post administration of DT(Scott et al., 2016).

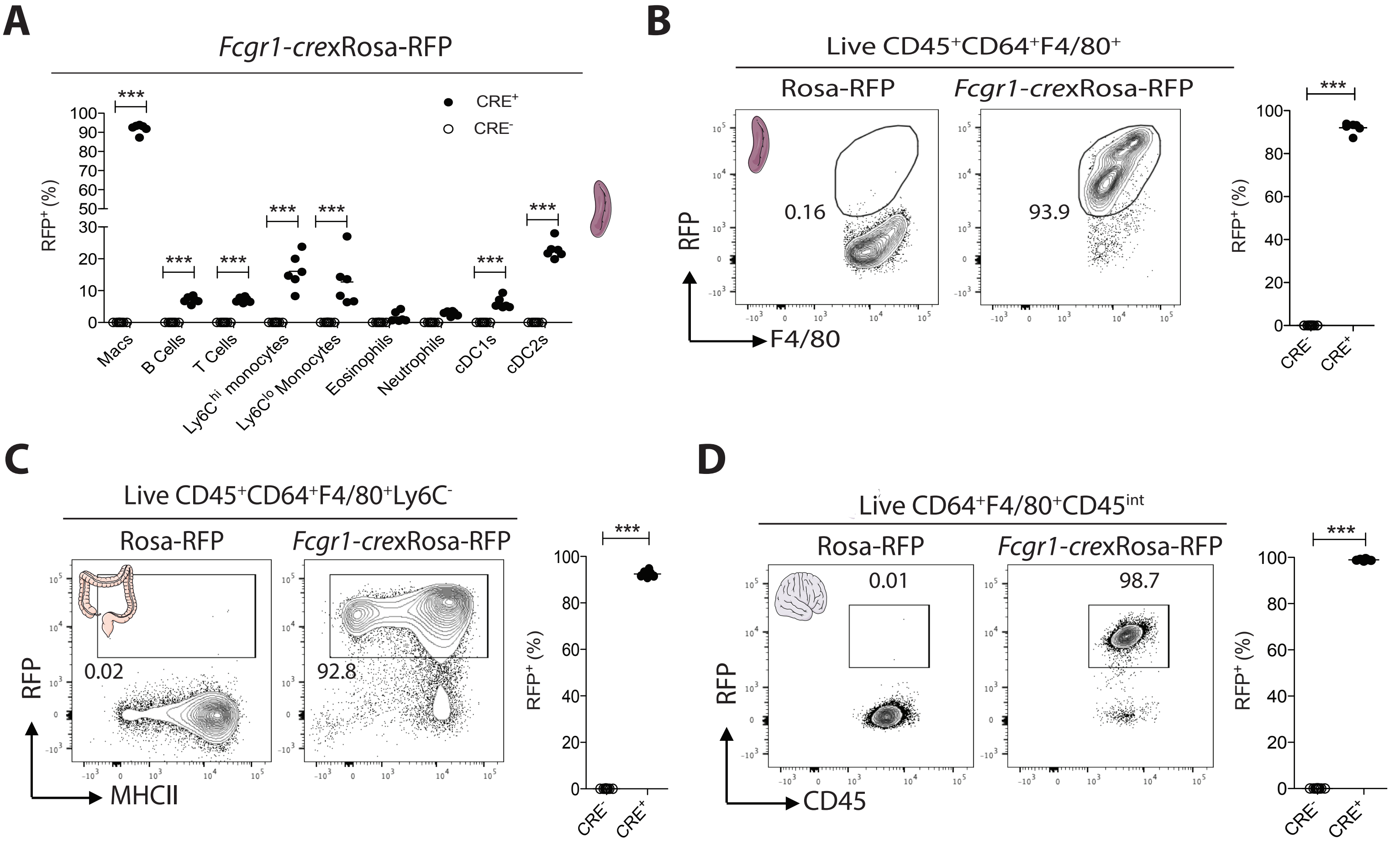
A
Fcgr1-crexRosa-RFP
CRE+
CRE-
RFP+ (%)
Macs
B Cells
T Cells
Ly6Chi monocytes
Ly6Clo Monocytes
Eosinophils
Neutrophils
cDC1s
cDC2s
B
Live CD45+CD64+F4/80+
Rosa-RFP
Fcgr1-crexRosa-RFP
0.16
93.9
RFP
F4/80
CRE-
CRE+
C
Live CD45+CD64+F4/80+Ly6C-
Rosa-RFP
Fcgr1-crexRosa-RFP
0.02
92.8
MHCII
D
Live CD64+F4/80+CD45int
Rosa-RFP
Fcgr1-crexRosa-RFP
0.01
98.7
CD45

**Figure S5; related to Figure 5: *Fcgr1-cre* validation.**

(A) Expression of RFP by indicated splenic immune cell populations including B cells, T cells, Neutrophils, Eosinophils and cDC1s and cDC2s (cDC subsets; defined on the basis of CD11b expression) in *Fcgr1-cre*xRosa-RFP$^{+/-}$ mice compared with Rosa-RFP$^{+/-}$ littermate controls. Data are pooled from 2 experiments with n= 6-9 per group. Students t test. ***p<0.001. (B) Expression of RFP by CD64$^{+}$F4/80$^{+}$Lin$^{-}$MHCII$^{-}$ splenic macs and % of splenic macs expressing RFP in *Fcgr1-cre*xRosaRFP$^{+/-}$ (CRE$^{+}$) and Rosa-RFP$^{+/-}$ (CRE$^{-}$) mice. Data are pooled from 2 experiments with n=6-9 per group. ***p<0.001 Student's t test. (C) Expression of RFP and MHCII by colonic macs in *Fcgr1-cre*xRosa-RFP$^{+/-}$ mice compared with Rosa-RFP$^{+/-}$ littermate controls. Data are pooled from 2 experiments with n= 6-9 per group. Students t test. ***p<0.001. (D) Expression of RFP and CD45 by microglia in *Fcgr1-cre*xRosa-RFP$^{+/-}$ mice compared with Rosa-RFP$^{+/-}$ littermate controls. Data are pooled from 2 experiments with n= 6-9 per group. Students t test. ***p<0.001.

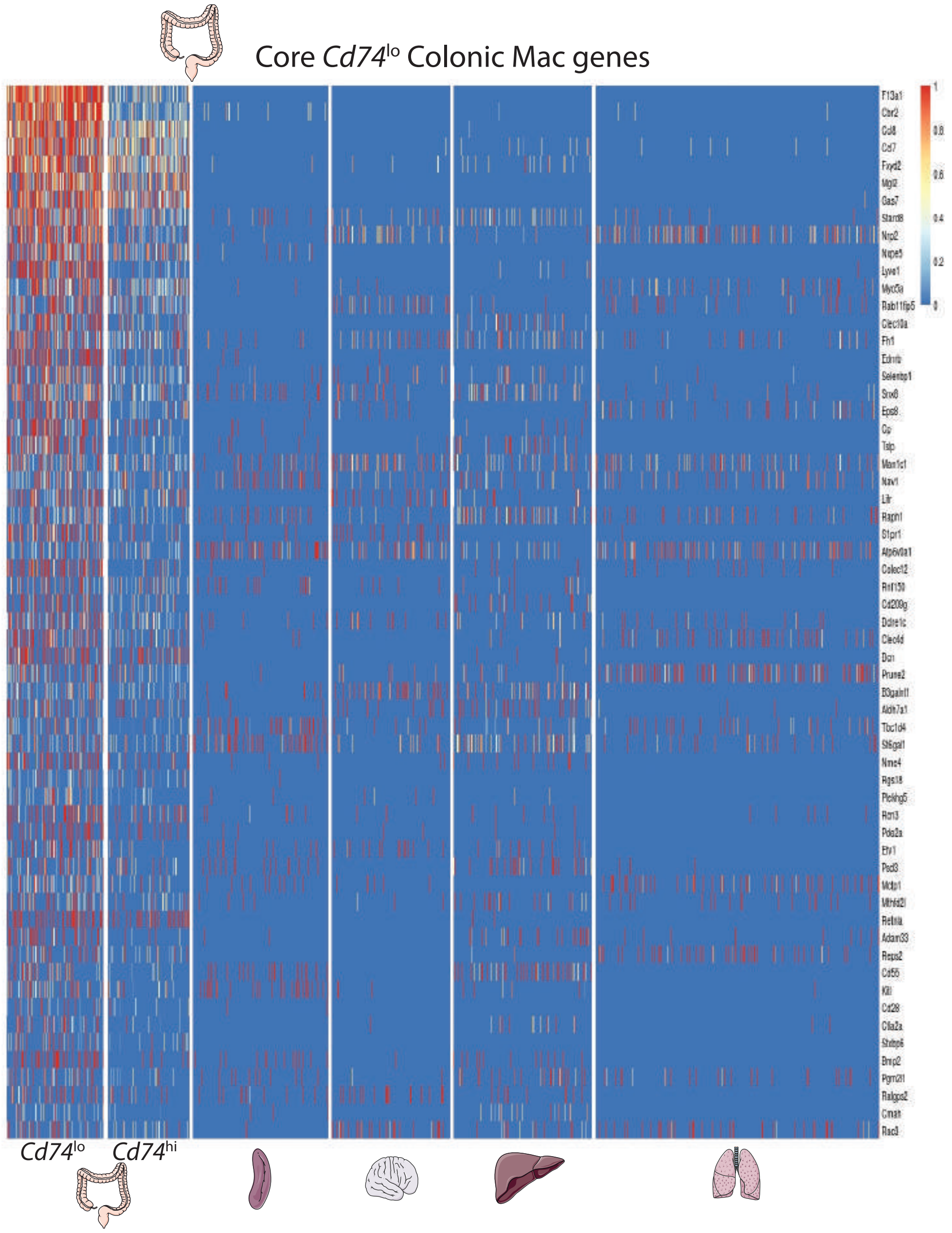
Core *Cd74*lo Colonic Mac genes
F13a1
Cbr2
Ccl8
Ccl7
Fxyd2
Mgl2
Gas7
Stard8
Nrp2
Napepld
Lyve1
Myo5a
Rab11fip5
Clec10a
Fh1
Ednrb
Selenbp1
Sox8
Eps8
Cp
Tslp
Man1c1
Nav1
Lifr
Raph1
S1pr1
Atp6v0a1
Colec12
Rnf150
Cd209g
Ddre1c
Clec4d
Dcn
Prune2
B3galnt1
Aldh7a1
Tbc1d4
St6gal1
Nme4
Rgs18
Plekhg5
Rcn3
Pde2a
Etv1
Psd3
Mctp1
Mthfd2l
Retnla
Adam33
Reps2
Cd55
Kit
Cd28
Cfla2a
Stxbp6
Bmp2
Pgm2l1
Ralgps2
Cmah
Rac3
1
0.8
0.6
0.4
0.2
0
*Cd74*lo
*Cd74*hi

**Figure S6; related to Figure 6: Core Transcriptomic profile of *Cd74*$^{lo}$ colonic macs**

Heatmap showing expression of core *Cd74*$^{lo}$ colonic mac genes. Heatmap was created by comparing SC-RNA-Seq data from *Cd74*$^{lo}$ colonic macs with *Cd74*$^{hi}$ colonic macs, KC, AMs, microglia and splenic macs.

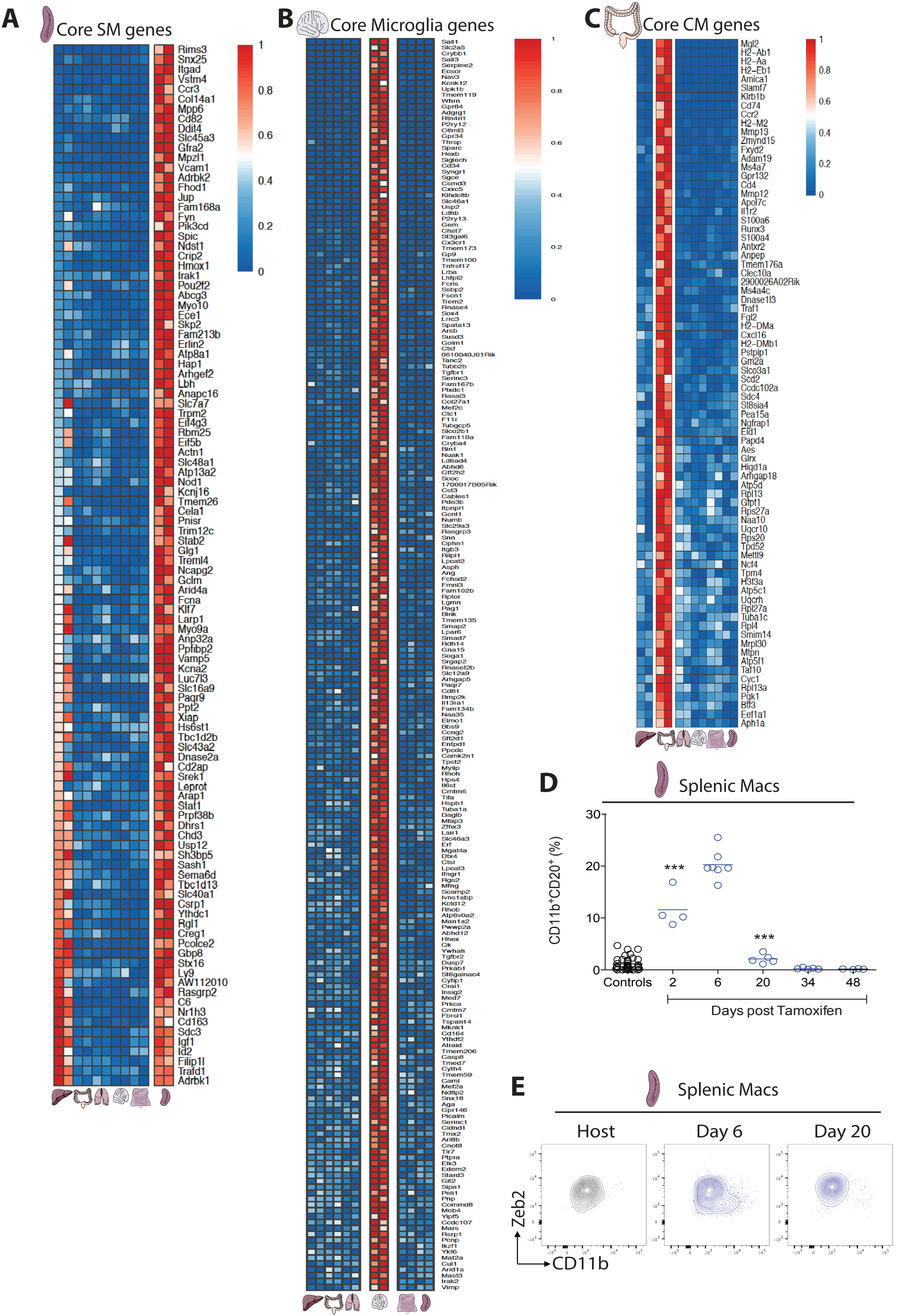

A
Core SM genes
Rims3
Snx25
Itgad
Vstm4
Ccr3
Col14a1
Mpp6
Cd82
Ddit4
Slc45a3
Gfra2
Mpzl1
Vcam1
Adrbk2
Fhod1
Jup
Fam168a
Fyn
Pik3cd
Spic
Ndst1
Crip2
Hmox1
Irak1
Pou2f2
Abcg3
Myo10
Ece1
Skp2
Fam213b
Erlin2
Atp8a1
Hap1
Arhgef2
Lbh
Anapc16
Slc7a7
Trpm2
Eif4g3
Rbm25
Eif5b
Actn1
Slc48a1
Atp13a2
Nod1
Kcnj16
Tmem26
Cela1
Pnisr
Trim12c
Stab2
Glg1
Treml4
Ncapg2
Gclm
Arid4a
Fcna
Klf7
Larp1
Myo9a
Anp32a
Ppfibp2
Vamp5
Kcna2
Luc7l3
Slc16a9
Paqr9
Ppt2
Xiap
Hs6st1
Tbc1d2b
Slc43a2
Dnase2a
Cd2ap
Srek1
Leprot
Arap1
Stat1
Prpf38b
Dhrs1
Chd3
Usp12
Sh3bp5
Sash1
Sema6d
Tbc1d13
Slc40a1
Csrp1
Ythdc1
Rgl1
Creg1
Pcolce2
Gbp8
Stx16
Ly9
AW112010
Rasgrp2
C6
Nr1h3
Cd163
Sdc3
Igf1
Id2
Filip1l
Trafd1
Adrbk1
1
0.8
0.6
0.4
0.2
0
B
Core Microglia genes
1
0.8
0.6
0.4
0.2
0
C
Core CM genes
Mgl2
H2-Ab1
H2-Aa
H2-Eb1
Amica1
Slamf7
Klrb1b
Cd74
Ccr2
H2-M2
Mmp13
Zmynd15
Fxyd2
Adam19
Ms4a7
Gpr132
Cd4
Mmp12
Apol7c
Il1r2
S100a6
Runx3
S100a4
Antxr2
Anpep
Tmem176a
Clec10a
2900026A02Rik
Ms4a4c
Dnase1l3
Traf1
Fgl2
H2-DMa
Cxcl16
H2-DMb1
Pstpip1
Gm2a
Slco3a1
Scd2
Ccdc102a
Sdc4
St8sia4
Pea15a
Ngfrap1
Eid1
Papd4
Aes
Glrx
Higd1a
Arhgap18
Atp5d
Rpl13
Gfpt1
Rps27a
Naa10
Uqcr10
Rps20
Tpd52
Mettl9
Ncf4
Tpm4
H3f3a
Atp5c1
Uqcrh
Rpl27a
Tuba1c
Rpl4
Smim14
Mrpl30
Mtpn
Atp5f1
Taf10
Cyc1
Rpl13a
Pgk1
Btf3
Eef1a1
Aph1a
1
0.8
0.6
0.4
0.2
0
D
Splenic Macs
CD11b+CD20+ (%)
30
20
10
0
***
***
Controls
2
6
20
34
48
Days post Tamoxifen
E
Splenic Macs
Host
Day 6
Day 20
Zeb2
CD11b

**Figure S7; related to Figure 7: Core transcriptomic profiles of splenic macs, microglia and colonic *Cd74*$^{hi}$ macs redefined and loss of *Zeb2*$^{-/-}$ splenic macs with time**

(A-C) Heatmap showing expression of core splenic mac (A), microglia (B) or colonic *Cd74*$^{hi}$ mac (C) genes by KCs, colonic macs, AMs, microglia, peritoneal macs and splenic macs. Data was previously published by (Lavin et al., 2014) and downloaded from the NCBI and filtered based on SC-RNA-Seq data to remove contaminating genes. (D) % CD11b$^{+}$CD20$^{+}$ splenic macs amongst total CD45.2$^{+}$ splenic macs at indicated time points (days) post the last dose of tamoxifen. Data are pooled from 2 experiments with n= 4-7 per time-point. ***$p<0.001$, One way ANOVA with Bonferroni post-test comparing each time point with the previous time point. (E) *Zeb2* mRNA expression (PrimeFlow) and CD11b expression in CD45.2 donor Splenic macs (blue) and CD45.1 host splenic macs (black) at indicated time-points.
